# NusA directly interacts with antitermination factor Q from phage λ

**DOI:** 10.1038/s41598-020-63523-5

**Published:** 2020-04-20

**Authors:** Benjamin R. Dudenhoeffer, Jan Borggraefe, Kristian Schweimer, Stefan H. Knauer

**Affiliations:** 1 Biopolymers, University of Bayreuth, Universitätsstraße 30, 95447 Bayreuth, Germany; 2 Present Address: Institute of Physical Biology, Heinrich-Heine-University, Universitätsstraße 1, 52428 Jülich, Germany; 3 Present Address: Institute of Complex Systems, Forschungszentrum Jülich, Wilhelm-Johnen-Straße, 52428 Jülich, Germany

**Keywords:** Solution-state NMR, Transcription, Biophysical chemistry

## Abstract

Antitermination (AT) is a ubiquitous principle in the regulation of bacterial transcription to suppress termination signals. In phage λ antiterminator protein Q controls the expression of the phage’s late genes with loading of λQ onto the transcription elongation complex halted at a σ-dependent pause requiring a specific DNA element. The molecular basis of λQ-dependent AT and its dependence on N-utilization substance (Nus) A is so far only poorly understood. Here we used solution-state nuclear magnetic resonance spectroscopy to show that the solution structure of λQ is in agreement with the crystal structure of an N-terminally truncated variant and that the 60 residues at the N-terminus are unstructured. We also provide evidence that multidomain protein NusA interacts directly with λQ via its N-terminal domain (NTD) and the acidic repeat (AR) 2 domain, with the λQ:NusA-AR2 interaction being able to release NusA autoinhibition. The binding sites for NusA-NTD and NusA-AR2 on λQ overlap and the interactions are mutually exclusive with similar affinities, suggesting distinct roles during λQ-dependent AT, e.g. the λQ:NusA-NTD interaction might position NusA-NTD in a way to suppress termination, making NusA-NTD repositioning a general scheme in AT mechanisms.

## Introduction

Transcription of all cellular genomes is mediated by evolutionary related multisubunit RNA polymerases (RNAPs)^1^. RNA synthesis is a discontinuous process that underlies tight regulation by various transcription factors that bind to RNAP, affecting its processivity. In Gram-negative bacteria the core RNAP consists of five subunits (2 x α, β, β’, ω). The flap region of the β subunit forms the outer wall of the RNA exit channel with the β flap tip helix (βFTH) at the top of this region being able to regulate the width of the channel, making it a key regulatory element^2–8^.

Antitermination (AT) is a ubiquitous mechanism to suppress termination signals and is widely used in bacteria. AT has first been described for bacteriophage λ where it controls the expression of early and late genes, being thus essential for the life cycle of the phage^9^. Phage λ uses two AT mechanisms which involve either antiterminator protein N or antiterminator protein Q. In λN-dependent AT the intrinsically disordered protein N is recruited to elongating RNAP by an AT signal in the nascent RNA and forms a complex with RNAP and the *Escherichia coli* (*E. coli*) host factors N-utilization substances (Nus) A, B, E, and G^6,7^. In this transcription AT complex (TAC) λN repositions NusA and remodels the βFTH, enabling the TAC to read through termination signals by preventing the formation of pause/terminator hairpins^6,7^.

In λQ-dependent AT protein λQ requires a λQ binding element (QBE) on the DNA for recruitment and is loaded onto RNAP halted at an adjacent sigma-dependent promoter-proximal pause site^10,11^. Recent cryo electron microscopy (EM) studies of the AT mechanism of protein Q from phage 21, Q21, revealed that two Q21 proteins engage with RNAP in a Q21-TAC, one of which forms a torus at the RNA exit channel, narrowing it to prevent the formation of pause/terminator hairpins^12,13^. Currently, over 15,000 lambdoid bacteriophage Q proteins have been identified, which can be grouped into three families (Q21 family: Pfam PF06530, λQ family: Pfam PF03589, and Q82 family: Pfam PF06323), and crystal structures of Q21 and an N-terminally truncated variant of λQ are available^12–14^. Although all Q proteins are encoded by genes located at equivalent positions in bacteriophage genomes and perform the same regulatory function they show neither significant amino acid sequence nor significant 3D structural similarity to each other, suggesting that the mechanism by which they mediate AT may be different.

AT does not only play a role in the life cycle of bacteriophages but is necessary for the expression of certain bacterial genes^15,16^. In ribosomal AT, for example, RNAP pauses at an AT signal and a TAC is formed that contains Nus factors A, B, E, and G as well as further components such as ribosomal protein S4 and inositol monophosphatase SuhB^17–20^. If this TAC is only responsible for AT or also involved in posttranscriptional activities, e.g. RNA maturation, is yet unclear.

Thus, Nus factors are key players in transcription regulation with NusG being a representative of the only class of transcription factors that is conserved in all three kingdoms of life^21^. *E. coli* NusG consists of two flexibly connected domains, an N-terminal and a C-terminal domain (NTD, CTD), respectively (Supplementary Fig. 1a)^22^. NusG-NTD has a mixed α/β topology and binds to the β’ clamp helices of RNAP, increasing its processivity, whereas NusG-CTD forms a five-stranded β-barrel that can interact with different binding partners (reviewed in^23^). For example, NusG-CTD is able to bind termination factor Rho, resulting in stimulation of Rho-dependent termination^24–26^, or to ribosomal protein S10 so that NusG serves as physical linker between RNAP and the ribosome to couple transcription and translation^24^. S10 consists of one domain with mixed α/β topology and is a moonlighting protein, i.e. it is identical to transcription factor NusE, if it is not part of the ribosome (Supplementary Fig. 1b). In this case NusE forms a heterodimer with NusB (Supplementary Fig. 1b), an α-helical, one-domain protein. During λN-dependent AT the NusE:NusB complex recognizes specifically a *boxA* element on the RNA with both NusE and NusB making contacts to the RNA^27,28^. NusE is the active component in processive AT whereas NusB supports loading of NusE to the transcription machinery^27^. NusG-CTD can interact with NusE and thus anchors the NusE:NusB:*boxA* complex to the RNAP^6,7,24^. NusG-mediated tethering of NusE:NusB:*boxA* to the RNAP might also be important for ribosomal AT^17,18^. The multidomain protein NusA (Supplementary Fig. 1c) is highly conserved in bacteria and regulates pausing and termination^2,29^, AT processes^30–32^, and RNA folding^33^. NusA-NTD binds to the βFTH^2,6,7^ and to the CTD of one of the RNAP α subunits (αCTD)^2^, whereas the following three domains (S1, K homology (KH)1, KH2) form the compact RNA binding motif SKK^34^. In *E. coli* and other γ-proteobacteria NusA has two acidic repeat (AR) domains, AR1 and AR2, at its C-terminus^35^. NusA-AR1 interacts with λN^36–38^, whereas NusA-AR2 serves as recruitment platform for various transcription factors such as the αCTD^39^, NusG-NTD^40^, and SuhB^17,18^. NusA is regulated via autoinhibition as NusA-AR2 binds to the KH1 domain, preventing RNA binding by NusA-SKK^39,41,42^. This autoinhibition may be released by RNAP αCTD to activate RNA binding by NusA^39^, by SuhB during ribosomal AT^17,18^ or by NusG-NTD, a process that might be play a role during NusG recruitment, in resynchronization of transcription:translation coupling or in modulation of termination efficiency^40^.

Nus factors are involved in various AT mechanisms. It has been demonstrated that NusA is able to stimulate Q-dependent AT in phage 82^43^ and it has been suggested that NusA promotes recruitment of Q in phage λ^32^. Here, we used solution-state nuclear magnetic resonance (NMR) spectroscopy to provide conclusive evidence that of all Nus factors only NusA interacts directly with λQ, establishing interactions *via* its NTD and AR2 domain. As NusA-NTD and λQ share binding sites on RNAP, the NusA-NTD:λQ interaction might be responsible for repositioning of NusA, stimulating λQ-mediated AT. We show that binding of λQ to NusA-AR2 releases autoinhibition of NusA, implying that NusA-AR2 is not only a versatile interaction platform for various transcription partners, but suggesting that NusA may be regarded as regulatory subunit of RNAP that substitutes the sigma factor.

## Results

### The solution structure of λQ

**Figure 1:**
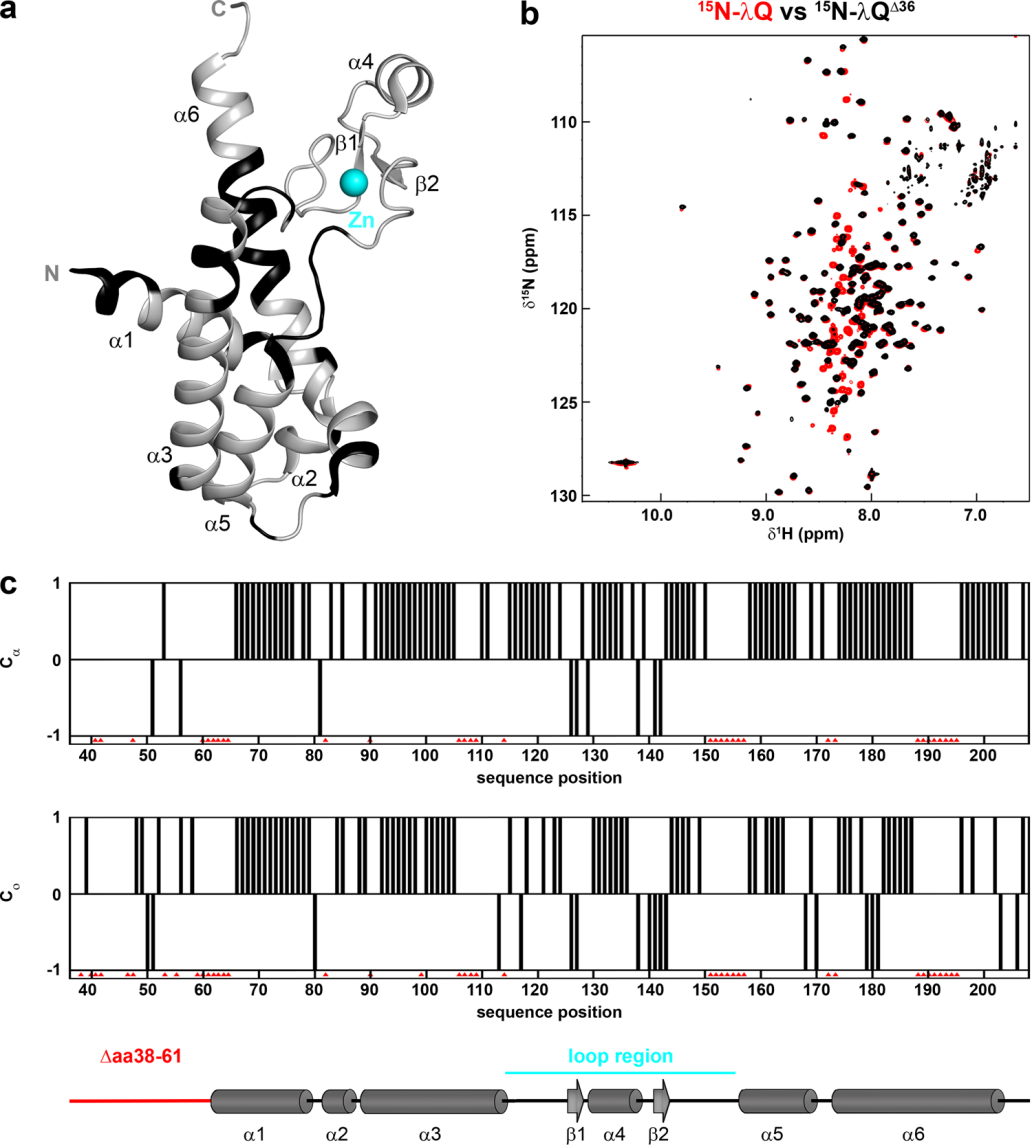
Structure of λQ^Δ36^. (**a**) Ribbon representation of the crystal structure of a λQ truncation variant lacking 38 residues at the N-terminus, electron density was only interpretable starting at residue 62 (PDB ID: 4MO1; gray). Termini and secondary structure elements are labeled. Unassigned amide backbone signals are shown in black, the Zn^2+^ is highlighted as cyan sphere. The PyMOL Molecular Graphics System (Version 1.7, Schrödinger, LLC.; https://pymol.org) was used for visualization. **(b)** Overlay of [^1^H,^15^N]-BEST-TROSY spectra of ^15^N-λQ (red) and ^15^N-λQ^Δ36^ (black). **(c)** Chemical shift index (CSI) for C_α_ and C_O_ of λQ^Δ36^ *vs*. secondary structure of the crystal structure (PDB ID: 4MO1). The missing residues due to non-interpretable electron density are indicated in red, the loop region is shown in cyan.

The 60 residues at the N-terminus of λQ are highly polar and are suggested to be disordered as, until now, only the crystal structure of a λQ variant lacking 38 residues at the N-terminus could be determined and even the electron density of this deletion variant was only interpretable starting at residue 62 (Fig. 1a)^14^. As the N-terminus, however, might have a functional role we used solution-state NMR spectroscopy to study the full length protein. The [^1^H,^15^N]-band-selective excitation short-transient transverse relaxation optimized spectroscopy (BEST-TROSY) spectrum of ^15^N-labeled λQ showed good signal dispersion (Fig. 1b) and perfectly superimposes with the spectrum of a λQ deletion variant lacking 36 residues at the N-terminus (λQ^Δ36^). Additional signals in the spectrum of the full length protein are all located between 7.5 and 8.5 ppm in the proton dimension, typical for random coil structures, suggesting that the N-terminus is indeed disordered in solution (Fig. 1b). Using λQ^Δ36^ we assigned 75.4% of the amide backbone and 78.4% of the C^α^ backbone signals (Fig. 1a). Unassigned residues are especially located at the N-terminus, at the C-terminal end of helix α3 (aa 108–120) and in the center of helix α6 (aa 189–198). The chemical shift index (CSI) of C_α_ and C_O_ atoms is in perfect agreement with the crystal structure (Fig. 1c), demonstrating that the three-dimensional (3D) structures in solution and in the crystal are identical. Analytical gel filtration showed that protein solutions of λQ and λQ^Δ36^ were homogeneous, giving molecular weights of 30 kDa and 17 kDa for λQ and λQ^Δ36^, respectively (theoretical molecular mass: 23 kDa and 16 kDa), indicating that both proteins exist as monomers in solution (Supplementary Fig. 2a). Next, we determined the ^15^N relaxation behavior of λQ^Δ36^ by NMR spectroscopy at 16.8 T magnetic field strength (Supplementary Fig. S2b) to characterize the overall tumbling of the protein. We obtained averaged ^15^N relaxation rates of 0.84 ± 0.06 s^−1^ and 19.5 ± 0.6 s^−1^ for *R*_1_ and *R*_2_, respectively. Assuming isotropic tumbling, these rates correspond to a rotation correlation time τ_c_ of 14 ± 1 ns, which suggests a molecular weight of ~23 kDa. This result supports the conclusion that the protein is a monomer in solution. The fact that the experimentally determined molecular weights of λQ and λQ^Δ36^ are slightly larger than the theoretical values for the monomeric proteins might be attributed to the facts that (i) both have an unstructured N-terminus (60 and 30 amino acids, respectively) and (ii) both have an elongated shape, i.e. both do not behave like perfectly globular proteins.

### λQ directly interacts with NusA

Nus factors are involved in both **λ**N-dependent and ribosomal AT. They are part of huge nucleoprotein complexes that modify RNAP into a termination-resistant state, so-called TACs^6,7,17,18,20^. Efficient **λ**Q-dependent AT requires at least NusA^44^, which is thought to stabilize binding of **λ**Q to the TEC^32^. Thus, we used solution-state NMR spectroscopy to test if λQ directly interacts with any of the Nus factors. We titrated ^15^N-labeled λQ with individual Nus factors and recorded one-dimensional (1D) [^1^H,^15^N]-heteronuclear single quantum coherence (HSQC) and two-dimensional (2D) [^1^H,^15^N]-BEST-TROSY spectra (Supplementary Fig. 3). For NusE we employed a protein variant which lacks the ribosome binding loop (NusE^Δ^), and which is in complex with NusB to increase solubility^27^. Only the presence of NusA (54.9 kDa) led to a significant change in the spectra of ^15^N-λQ (Supplementary Fig. 3), namely the signal intensity of ^15^N-λQ was significantly decreased upon addition of NusA (Supplementary Fig. 3a). Slower tumbling leads to increased transverse relaxation rates, which results in line broadening and thus ultimately in a decrease of signal intensity of ^15^N-λQ signals. Consequently, the loss of ^15^N-λQ signal intensity suggests a direct λQ:NusA interaction.

### λQ binds to NusA-NTD

**Figure 2:**
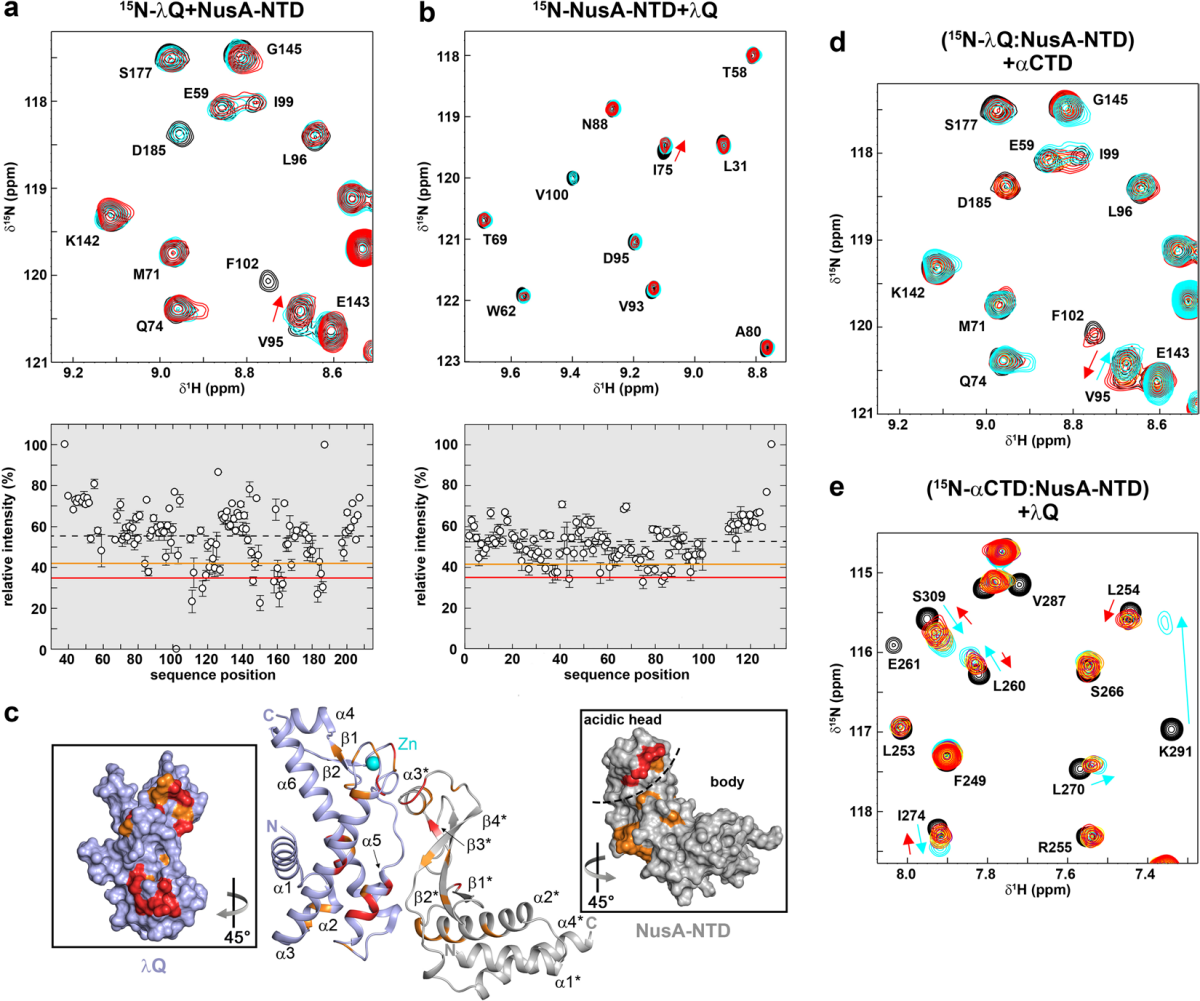
NusA interacts with λQ via its NTD. (a) (top) Section of [^1^H,^15^N]-BEST-TROSY spectra of the titration of 250 µM ^15^N-λQ with NusA-NTD (molar ratios: 1:0, black; 1:1, cyan; 1:2 red). Selected signals are labeled. (bottom) Relative intensity of ^15^N-λQ signals in the presence of one equivalent NusA-NTD. Orange and red lines indicate thresholds for moderately (1.0 σ of average relative signal intensity) and strongly (1.5 σ of average relative signal intensity) affected signals, respectively. The dashed line represents the mean relative intensity, error bars are given as black vertical lines. **(b)** (top) Section of [^1^H,^15^N]-HSQC spectra of the titration of 175 µM ^15^N-NusA-NTD with λQ (molar ratios: 1:0, black; 1:1, cyan; 1:2 red). Selected signals are labeled. (bottom) Relative intensity of ^15^N-NusA-NTD signals in the presence of one equivalent λQ with error bars. Thresholds as in (a). **(c)** Model of the λQ:NusA-NTD complex. The model was generated with the HADDOCK 2.2 server (https://haddock.science.uu.nl/services/HADDOCK2.2/) using affected residues as determined via NMR spectroscopy as restraints (see Supplementary Table 1). The model with the lowest HADDOCK and Z-score is depicted. λQ (PDB ID: 4MO1, lightblue) and NusA-NTD (PDB ID: 2KWP, gray) are shown in ribbon representation. Affected residues are colored (moderately affected residues, orange; strongly affected residues, red). Termini and secondary structure elements are labeled. Panels show the surface representations of λQ (left) and NusA-NTD (right) colored as in the complex. The PyMOL Molecular Graphics System (Version 1.7, Schrödinger, LLC.; https://pymol.org) was used for visualization. **(d)** αCTD detaches NusA-NTD from ^15^N-λQ. Sections of [^1^H,^15^N]-BEST-TROSY spectra are shown. Molar ratios: ^15^N-λQ:NusA-NTD: αCTD = 1:0:0, black; =1:2:0, cyan; 1:2:2, orange; 1:2:4, red. Initial concentration of ^15^N-λQ: 250 µM. Selected signals are labeled, arrows indicate chemical shift changes upon addition of NusA-NTD (cyan) and αCTD (red). **(e)** λQ removes NusA-NTD from ^15^N-αCTD. Sections of [^1^H,^15^N]-HSQC spectra are shown. Molar ratios: ^15^N-αCTD:NusA-NTD:λQ = 1:0:0; black; =1:2:0; cyan; 1:2:2; orange; 1:2:4, purple; 1:2:6, yellow; 1:2:10, red. Initial concentration of ^15^N-αCTD: 250 µM. Selected signals are labeled, arrows indicate chemical shift changes upon addition of NusA-NTD (cyan) and λQ (red).

In order to identify the region of NusA that binds to λQ we carried out [^1^H,^15^N]-HSQC-based titrations of λQ and the individual domains of NusA with 1D and 2D spectra being recorded after each titration step. Titration of ^15^N-λQ with NusA-NTD led to a significant change of the ^15^N-λQ spectrum (Fig. 2a and Supplementary Fig. 4a), indicating complex formation. Likewise ^15^N-NusA-NTD signals were substantially affected in the presence of λQ (Fig. 2b and Supplementary Fig. 4b). In both titrations the signal intensity of the labeled protein was decreased non-uniformly upon addition of the binding partner whereas chemical shift perturbations were only small, suggesting intermediate or slow chemical exchange on the NMR time scale. Thus, in both titrations the change of signal intensity was analysed quantitatively by calculating the relative signal intensity of the ^15^N-labeled protein in the presence of one equivalent of the non-labeled binding partner (for details see Material and Methods). In brief, we defined the relative intensity as ratio of the normalized remaining signal intensity of the ^15^N-labeled protein in the presence of the binding partner to the normalized signal intensity of the free labeled protein. The relative intensity was plotted against the corresponding amino acid position (Fig. 2a,b) and thresholds at 1.5 σ and 1.0 σ of the mean relative signal intensity were used to identify strongly and moderately affected residues, respectively, which were then mapped onto the 3D structures of λQ and NusA-NTD (Fig. 2c). λQ residues affected by NusA-NTD binding form two continuous patches. The first patch involves residues located in α3 and α5, the second patch contains residues at C- and N-terminal parts of the Zn-binding motif (Fig. 2c, left inset). NusA-NTD residues affected by binding of λQ are primarily located in the acidic head region and the upper part of the body region at the convex side of NusA-NTD. Due to insufficient long-term stability we did not record intermolecular nuclear Overhauser effect (NOE) interactions. Thus a docking model was generated based on the affected residues in both proteins and without allowing conformational rearrangements (Supplementary Fig. 4c-e). In the lowest energy model (Fig. 2c) the acidic head of NusA-NTD contacts the Zn-finger of λQ and the upper part of the NusA-NTD body interacts with the second patch of λQ, giving an overall interface area of 1050 Å².

We also checked if the unstructured N-terminus of λQ is involved in NusA-NTD binding by titrating ^15^N-NusA-NTD with λQ^Δ36^ and recorded a 2D [^1^H,^15^N]-HSQC spectrum after each titration step (Supplementary Fig. 5a). Like in the titration with full length λQ the intensity of ^15^N-NusA-NTD signals was significantly decreased in the presence of λQ^Δ36^, and the relative signal intensity in the equimolar titration step was plotted against the amino acid position of NusA-NTD (Supplementary Fig. 5b). Mapping of the affected residues onto the NusA-NTD structure (Supplementary Fig. 5c) resulted in the same binding site as identified in the titration with full length λQ, indicating that the N-terminus of λQ does not influence the interaction with NusA-NTD.

### λQ and RNAPαCTD share binding sites on NusA-NTD

NusA-NTD contacts two regions of RNAP, the βFTH and the αCTD^2^. The λQ binding site is located on the convex side of NusA-NTD, overlapping with the binding site for RNAP αCTD^2^, thus suggesting that binding of λQ and αCTD might be competitive. To test if the interactions of αCTD and λQ with NusA-NTD are mutual exclusive, we performed NMR-based competition experiments with [^1^H,^15^N]-HSQC spectra being recorded after each titration step. First, NusA-NTD was added in a two-fold molar excess to ^15^N-Q, resulting in signal changes typical for ^15^N-λQ:NusA-NTD complex formation (Fig. 2d and Supplementary Fig. 6a; see also Fig. 2a). Subsequent titration with αCTD reversed the signal changes (both chemical shift changes and loss of signal intensity), demonstrating that αCTD binds to NusA-NTD, detaching it from λQ. To confirm this finding we carried out a reverse experiment where NusA-NTD was added to ^15^N-αCTD in a 1:2 molar ratio, leading to chemical shift perturbations that indicate complex formation. Addition of λQ reversed those changes, indicating that λQ removes NusA-NTD from αCTD by complexing it (Fig. 2e and Supplementary Fig. 6b). These results imply that the λQ:NusA-NTD and αCTD:NusA-NTD interactions are mutually exclusive and have similar affinities, which, in turn, suggests that both interactions are physiologically relevant. We excluded a direct αCTD:λQ interaction as the titration of ^15^N-λQ with αCTD did not alter the spectra of ^15^N-λQ (Supplementary Fig. 6c).

### λQ does not interact with NusA-SKK and NusA-AR1

Having identified NusA-NTD as interaction partner of λQ we tested next if also NusA-SKK binds to it. We titrated ^15^N-labeled λQ with NusA-SKK and *vice versa* and recorded 1D- and 2D-[^1^H,^15^N] correlation spectra after each titration step (Supplementary Fig. 7a,b). Even in the presence of a twofold molar excess of the unlabeled binding partner no significant changes were observable in the spectra of the labeled protein, excluding a direct NusA-SKK:λQ interaction. Using the same approach we asked if λQ binds to NusA-AR1, which contacts λN in λN-dependent AT^36–38^, but again, no direct interaction could be detected (Supplementary Fig. 7c,d).

### λQ binds to NusA-AR2

**Figure 3:**
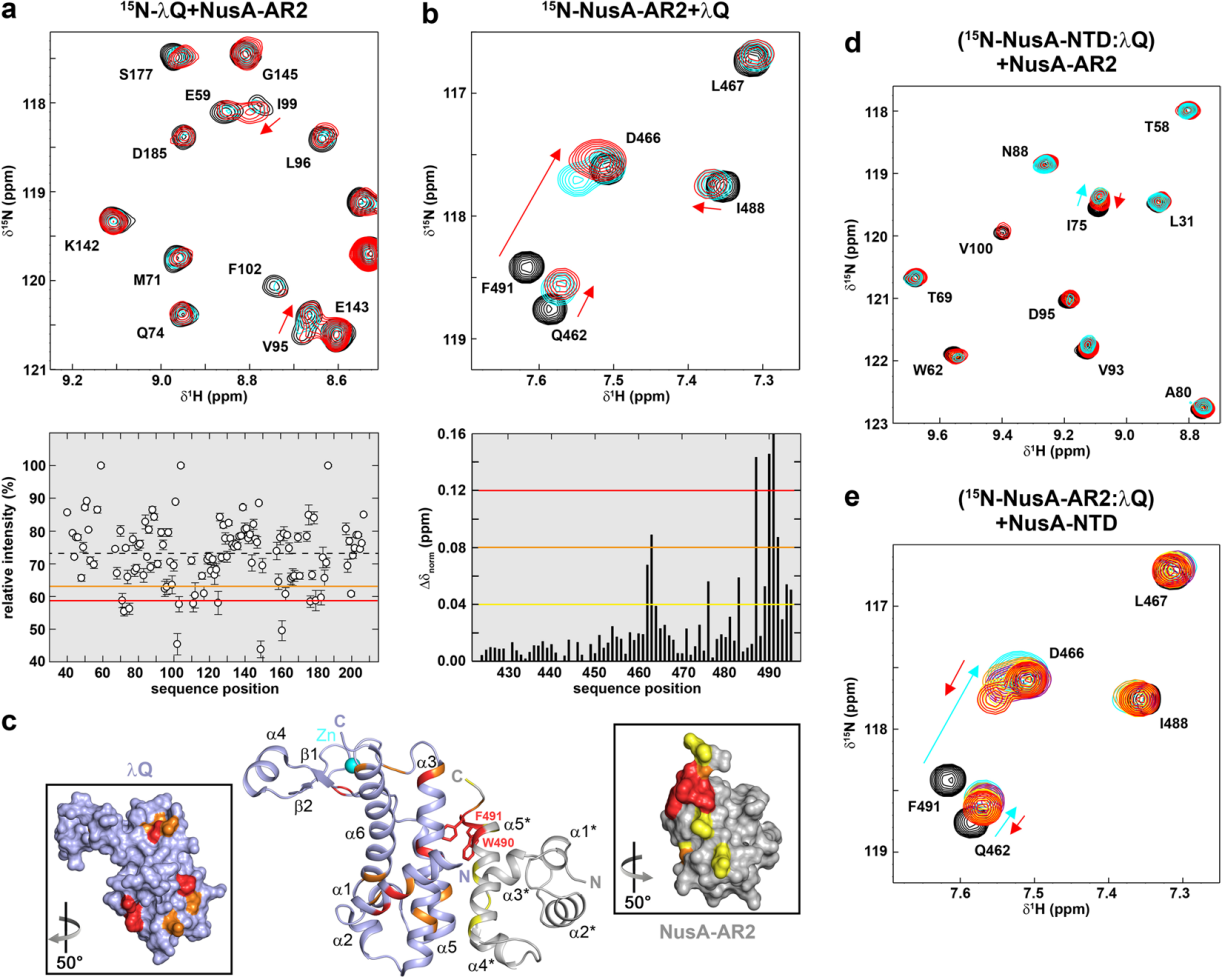
NusA interacts with λQ via its AR2 domain. (**a**) (top) Section of [^1^H,^15^N]-BEST-TROSY spectra of the titration of 250 µM ^15^N-λQ with NusA-AR2. NusA-AR2 (molar ratios: 1:0, black; 1:1, cyan; 1:2 red). Selected signals are labeled. (bottom) Relative intensity of ^15^N-λQ signals in the presence of two equivalents NusA-AR2. Error bars are given as black vertical lines. Orange and red lines indicate thresholds for moderately (1.0 σ of average relative signal intensity) and strongly (1.5 σ of average relative signal intensity) affected signals, respectively. The dashed line represents the mean relative intensity. **(b)** (top) Section of [^1^H,^15^N]-HSQC spectra of the titration of 250 µM ^15^N-NusA-AR2 with λQ (molar ratios: 1:0, black; 1:1, cyan; 1:2 red). Selected signals are labeled. (bottom) Normalized chemical shift changes of ^15^N-NusA-AR2 upon addition of two equivalents λQ. Yellow, orange, and red lines indicate thresholds for slightly (0.04 ppm ≤ *Δ*δ_norm_ < 0.08 ppm), moderately (0.08 ppm ≤ *Δ*δ_norm_ < 0.12 ppm) and strongly (*Δ*δ_norm_ ≥ 0.12 ppm) affected signals. **(c)** Model of the λQ:NusA-AR2 complex. The model was generated using the HADDOCK 2.2 server (https://haddock.science.uu.nl/services/HADDOCK2.2/) with the affected residues as determined by NMR spectroscopy as restraints (see Supplementary Table 1). The model with the lowest HADDOCK score is depicted. λQ (PDB ID: 4MO1, lightblue) and NusA-AR2 (PDB ID: 1WCN, gray) are in ribbon representation. Affected residues are colored (λQ: moderately affected residues, orange; strongly affected residues, red; NusA-AR2: slightly affected residues, yellow; moderately affected residues, orange; strongly affected residues, red). Termini and secondary structure elements are labeled. Panels show the surface representations of λQ (left) and NusA-AR2 (right) colored as in the complex. The PyMOL Molecular Graphics System (Version 1.7, Schrödinger, LLC.; https://pymol.org) was used for visualization. **(d)** NusA-AR2 removes λQ from ^15^N-NusA-NTD. Sections of [^1^H,^15^N]-HSQC spectra are depicted. Molar ratios: ^15^N-NusA-NTD:λQ:NusA-AR2 = 1:0:0, black; =1:2:0, cyan; 1:2:2, orange; 1:2:4, red. Initial concentration of ^15^N-NusA-NTD: 250 µM. Selected signals are labeled, arrows indicate the chemical shift changes upon addition of λQ (cyan) and NusA-AR2 (red). **(e)** NusA-NTD detaches λQ from ^15^N-NusA-AR2. Sections of [^1^H,^15^N]-HSQC spectra are shown. Molar ratios: ^15^N-NusA-AR2:λQ:NusA-NTD = 1:0:0; black; =1:2:0; cyan; 1:2:2; orange; 1:2:4, purple; 1:2:6, yellow; 1:2:10, red. Initial concentration of ^15^N-NusA-AR2: 250 µM. Selected signals are labeled, arrows show changes of chemical shifts upon addition of λQ (cyan) and NusA-NTD (red).

Finally, we tested if λQ interacts with NusA-AR2. Upon addition of NusA-AR2 to ^15^N-λQ the 1D and 2D spectra of ^15^N-λQ changed significantly (Fig. 3a and Supplementary Fig. 8a). As in the titration of ^15^N-λQ with NusA-NTD, the intensity of ^15^N-λQ signals decreased non-uniformly whereas only slight chemical shift perturbation were observable. Thus, we analyzed the change of signal intensity quantitatively. The relative intensity of ^15^N-λQ signals in the presence of two equivalents NusA-AR2 was plotted against the amino acid sequence of λQ and affected residues were identified by using thresholds at 1.5 and 1.0 σ of the mean relative intensity (Fig. 3a). In contrast, the titration of ^15^N-NusA-AR2 with λQ resulted in significant chemical shift perturbations and normalized chemical shift changes (Δδ_norm_) were plotted against the amino acid sequence of NusA-AR2 (Fig. 3b and Supplementary Fig. 8b). To visualize binding surfaces affected residues were mapped on the 3D structures of λQ and NusA-AR2 (Fig. 3c). λQ residues affected by NusA-AR2 binding are located opposite the Zn binding motif and the flexible arm, and strongly affected residues can be found predominantly in helices α3 and α5.

The λQ binding site of NusA-AR2 is located at the C-terminal part of the domain and comprises helix α5*, with W490 and F491 being strongly affected. These two residues are known to be responsible for the specific recognition of other transcription regulators such as αCTD^39^, NusG-NTD^40^, or SuhB^17,18^. As for the λQ:NusA-NTD complex, we did not record intermolecular NOE interactions due to insufficient long-term stability of λQ. Based on the identified binding surfaces a docking model of the λQ:NusA-AR2 complex was generated without allowing conformational rearrangements (Supplementary Fig. 8c-e). In the lowest energy model (Fig. 3c) helix α5* of NusA-AR2 packs against helices α3 and α5 of λQ so that W490 and F491 are central parts of the interface, which comprises a total area of 1230 Å². Finally, we determined the *K*_D_ for the λQ:NusA-AR2 complex by fluorescence anisotropy measurements using NusA-AR2^D443C^, a NusA-AR2 variant where D443, located opposite the λQ binding site, is substituted by a Cys^18^. NusA-AR2^D443C^ was labeled site-specifically with fluorescein5-maleimide and titrated with λQ, giving a *K*_D_ of 268 ± 17 µM (Supplementary Fig. 8f). Repeating the HSQC-based titration of ^15^N-NusA-NTD with λQ^Δ36^ resulted in the same binding surface as determined for full length λQ (Supplementary Fig. 8g-i), suggesting that the unstructured N-terminus of λQ is not involved in NusA-AR2 binding. This finding was corroborated by fluorescence anisotropy measurements as the affinity of the NusA-AR2^D443C^:λQ^Δ36^ interactions was determined to be 271 ± 27 µM (Supplementary Fig. 8j).

### NusA-NTD and NusA-AR2 share binding sites on λQ

In summary, λQ is able to establish interactions with NusA-NTD and NusA-AR2 and comparison of their binding sites on λQ suggests that they are partially overlapping, involving residues located in helices α3 and α5. To test if binding of NusA-NTD and NusA-AR2 is indeed competitive, we carried out 2D [^1^H,^15^N]-HSQC-based competition experiments with spectra being recorded after each titration step. First, λQ was added in a two-fold molar excess to ^15^N-NusA-NTD, resulting in changes of the ^15^N-NusA-NTD spectrum typical for ^15^N-NusA-NTD:λQ complex formation (Fig. 3d and Supplementary Fig. 9a). Subsequent titration with NusA-AR2 reversed those changes partially (Fig. 3d), demonstrating that NusA-AR2 complexes λQ, detaching it from NusA-NTD. To corroborate this finding we formed a ^15^N-NusA-AR2:λQ complex (molar ratio 1:2) leading to chemical shift perturbations of the ^15^N-NusA-AR2 signals that confirm λQ binding (Fig. 3e and Supplementary Fig. 9b). Upon addition of NusA-NTD the chemical shifts shifted back towards their position in the spectrum of free ^15^N-NusA-AR2 (Fig. 3e), showing that NusA-NTD binds to λQ while removing it from NusA-AR2. Thus, the λQ:NusA-NTD and the λQ:NusA-AR2 interactions are mutually exclusive with similar affinities.

### λQ and RNAPαCTD share binding sites on NusA-AR2

In free NusA the AR2 domain binds to the KH1 domain of the SKK motif, preventing RNA binding by NusA-SKK and rendering NusA autoinhibited^39,41,42^. This autoinhibition can be released by the αCTD of RNAP as NusA-SKK and αCTD share binding sites on NusA-AR2^39^. NusA-AR2, however, can also bind to NusG-NTD, an interaction that might be involved in the regulation of Rho-dependent termination or in the recruitment of NusG^40^, and to SuhB^17,18^. The NusA-AR2:SuhB complex formation is suggested to play a role in the transcriptional or posttranscriptional regulation of ribosomal AT. Interestingly, the binding sites for NusG-NTD, SuhB, and αCTD as well as λQ on NusA-AR2 all involve the C-terminal helix α5 and overlap. Moreover, it has been demonstrated that the interactions of NusA-AR2 with NusG-NTD, αCTD, and SuhB are competitive^18^.

**Figure 4:**
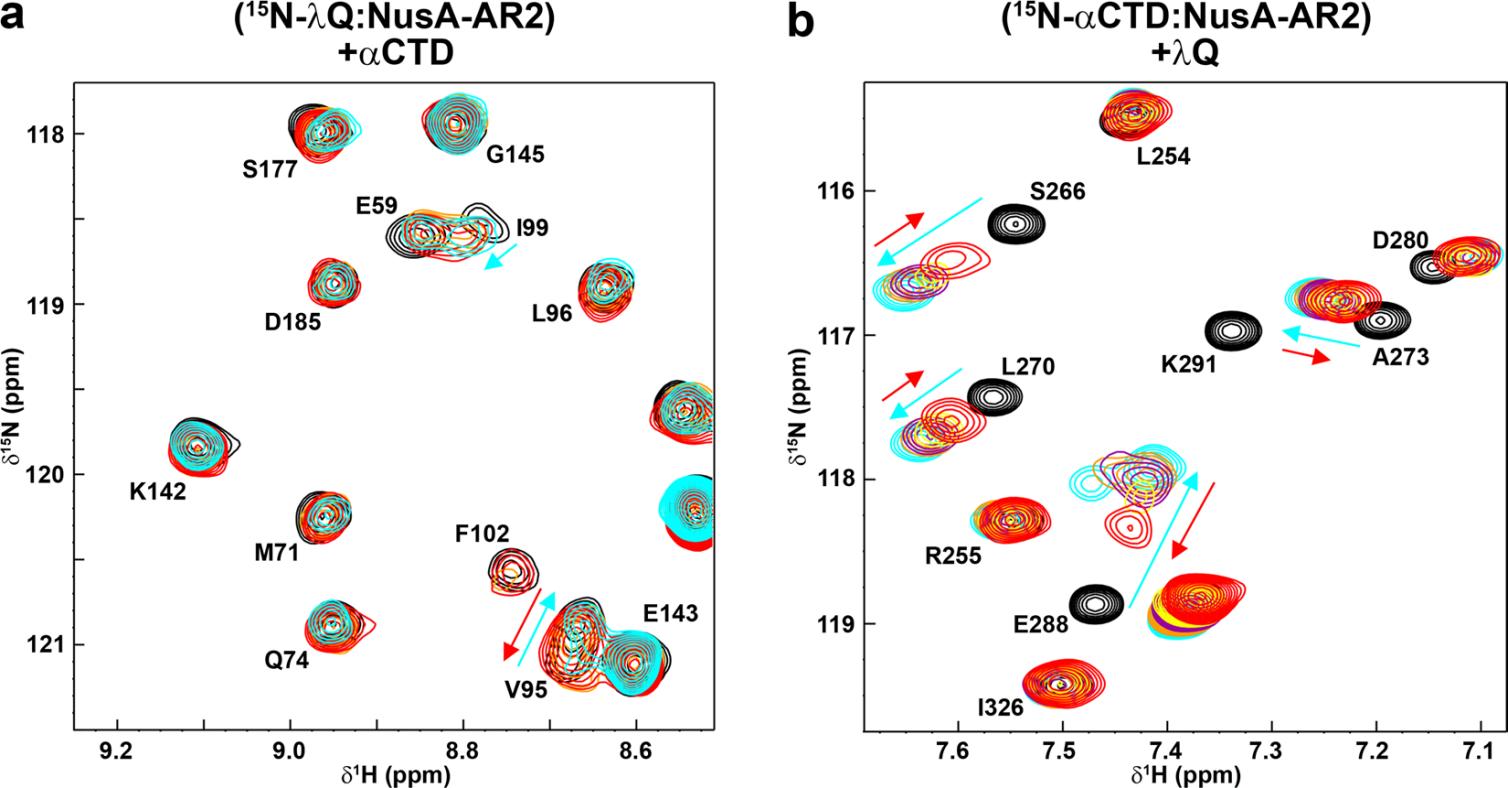
αCTD and λQ share binding sites on NusA-AR2. NMR-based competition experiments of λQ, αCTD, and NusA-AR2. **(a)** NusA-AR2 is detached from ^15^N-λQ by αCTD. Sections of [^1^H,^15^N]-BEST-TROSY spectra are shown. Molar ratios: ^15^N-λQ:NusA-AR2:αCTD = 1:0:0, black; =1:2:0, cyan; 1:2:2, orange; 1:2:4, red. Initial concentration of ^15^N-λQ: 250 µM. Selected signals are labeled, arrows indicate chemical shift changes upon addition of NusA-AR2 (cyan) and αCTD (red). **(b)** λQ removes NusA-AR2 partially from ^15^N-αCTD. Sections of [^1^H,^15^N]-HSQC spectra are depicted. Molar ratios: Molar ratios: ^15^N-αCTD:NusA-AR2:λQ = 1:0:0; black; =1:2:0; cyan; 1:2:2; orange; 1:2:4, purple; 1:2:6, yellow; 1:2:10, red. Initial concentration of ^15^N-αCTD: 250 µM. Selected signals are labeled and arrows show changes in chemical shifts upon addition of NusA-AR2 (cyan) and λQ (red).

To show that also λQ competes with NusG-NTD, αCTD, and SuhB for NusA-AR2 binding we tested if the complexes λQ:NusA-AR2 and αCTD:NusA-AR2 are mutually exclusive by 2D NMR-based competition experiments with spectra being recorded after each titration step. First, NusA-AR2 was added in a twofold molar excess to ^15^N-λQ, resulting in changes of the ^15^N-λQ spectrum corresponding to ^15^N-λQ:NusA-AR2 complex formation. Subsequent titration with the αCTD reversed both the chemical shift changes and the loss of signal intensity, showing that the αCTD detaches NusA-AR2 from λQ by binding to it (Fig. 4a and Supplementary Fig. 10a). To confirm this result we performed another competition experiment where λQ was titrated to a preformed ^15^N-αCTD:NusA-AR2 complex (molar ratio 1:2). The addition of λQ reversed partially the chemical shift perturbations caused by the ^15^N-αCTD:NusA-AR2 complex formation (Fig. 4b and Supplementary Fig. 10b), demonstrating that λQ can bind to NusA-AR2 in order to remove it from the αCTD. Together with previous data^18,40^ this finding leads to the conclusion that the NusA-AR2 binding sites for αCTD, λQ, NusG-NTD, and SuhB largely overlap, rendering the interactions of these binding partners with NusA-AR2 competitive. Finally, the *K*_D_ value of the αCTD:NusA-AR2 interaction was determined by fluorescence anisotropy measurements using the NusA-AR2^D443C^ variant to be 8±1 µM (Supplementary Fig. 10c), in agreement with a previous report^39^. Thus, the affinity of the NusA-AR2^D443C^:αCTD interaction is significantly higher than the one of the NusA-AR2^D443C^:λQ interaction, which explains why λQ is able to detach NusA-AR2 only partially from the αCTD (Fig. 4).

### λQ may release the autoinhibition of NusA

**Figure 5:**
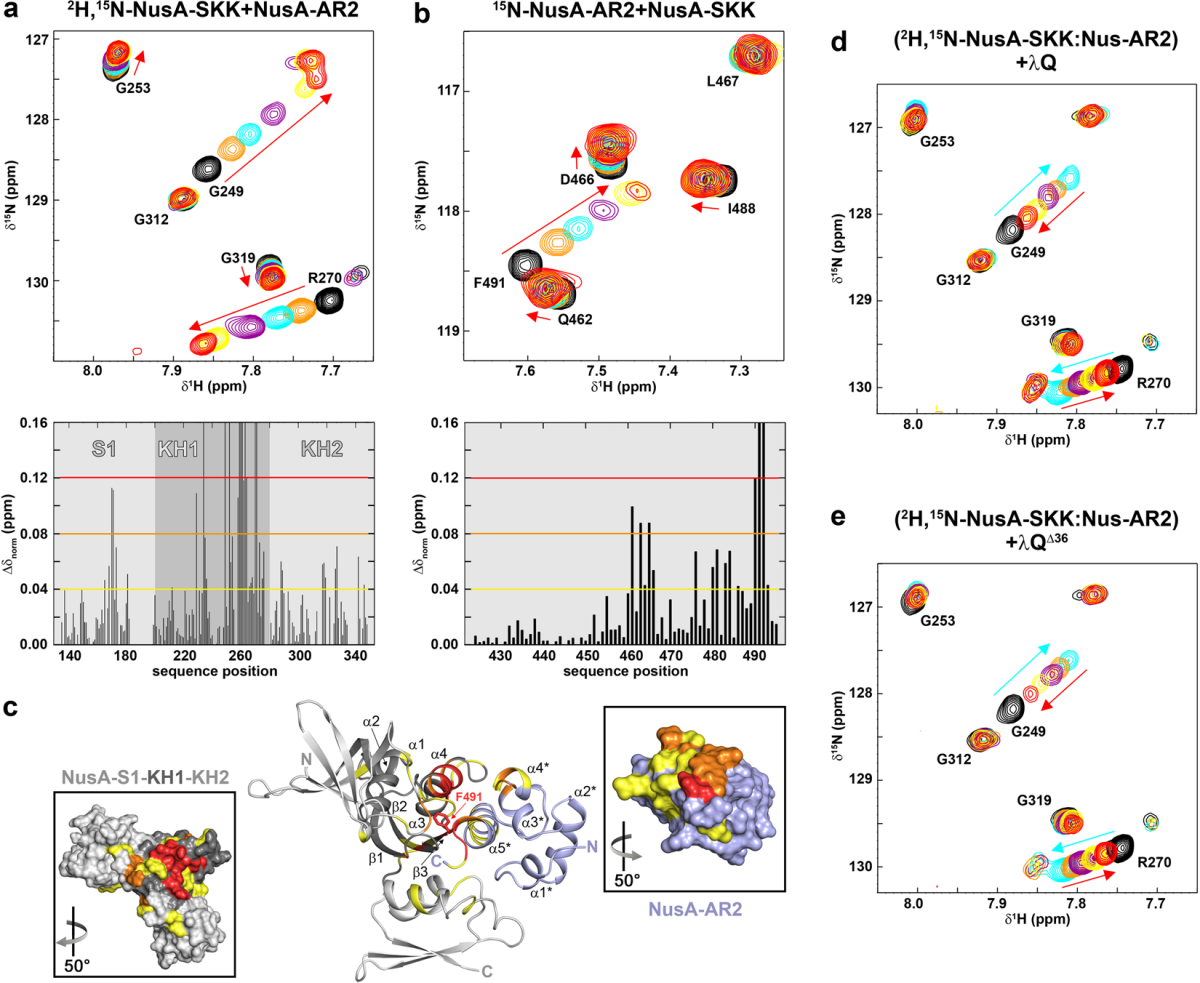
λQ releases autoinhibition of NusA. (**a**) (top) Sections of [^1^H,^15^N]-BEST-TROSY spectra of the titration of 300 µM ^2^H,^15^N-NusA-SKK with NusA-AR2 (molar ratios: 1:0, black; 1:0.5, orange; 1:1, cyan; 1:2 purple; 1:5 yellow; 1:7.5 red; stock concentration of NusA-AR2: 1.5 mM). Selected signals are labeled. (bottom) Normalized chemical shift perturbations of ^2^H,^15^N-NusA-SKK signals upon addition of 7.5 equivalents NusA-AR2. Yellow, orange, and red lines indicate thresholds for slightly (0.04 ppm ≤ *Δ*δ_norm_ < 0.08 ppm), moderately (0.08 ppm ≤ *Δ*δ_norm_ < 0.12 ppm) and strongly (*Δ*δ_norm_ ≥ 0.12 ppm) affected signals. **(b)** (top) Sections of [^1^H,^15^N]-HSQC spectra of the titration of 200 µM ^15^N-NusA-AR2 with NusA-SKK (molar ratios: 1:0, black; 1:0.5, orange; 1:1, cyan; 1:2 purple; 1:5 yellow; 1:7.5 red; stock concentration of NusA-SKK: 500 µM). Selected signals are assigned. (bottom) Normalized chemical shift changes of ^15^N-NusA-AR2 upon addition of 7.5 equivalents NusA-SKK. Thresholds as in **(b)**. **(c)** Model of the NusA-SKK:NusA-AR2 complex. The model was generated with the HADDOCK 2.2 server (https://haddock.science.uu.nl/services/HADDOCK2.2/) using affected residues as determined by NMR spectroscopy as restraints (see Supplementary Table 1). The model with the lowest HADDOCK score and Z-score is depicted. NusA-AR2 (PDB ID: 1WCN, lightblue) and NusA-SKK (PDB ID: 5LM9, gray) are in ribbon representation. Affected residues are colored (slightly affected residues, yellow; moderately affected residues, orange; strongly affected residues, red). Termini and secondary structure elements are labeled. Panels show the surface representations of NusA-AR2 (left) and NusA-SKK (right), colored as in the complex, whereby the KH1 domain is highlighted in dark gray. The PyMOL Molecular Graphics System (Version 1.7, Schrödinger, LLC.; https://pymol.org) was used for visualization. **(d)** λQ and **(e)** λQ^Δ36^ detach NusA-AR2 from ^2^H,^15^N-NusA-SKK. Sections of [^1^H,^15^N]-BEST-TROSY are shown. Molar ratios: ^2^H,^15^N-NusA-SKK: NusA-AR2:λQ/λQ^Δ36^ =1:0:0, black; =1^:^5:0, orange; =1:5:1, cyan; =1:5:2, purple; =1:5:5, yellow; =1:5:10, red. Initial concentration of ^2^H,^15^N-NusA-SKK: 175 µM. Selected signals are labeled and arrows indicate chemical shift changes upon addition of NusA-AR2 (cyan) and λQ/λQ^Δ36^ (red).

NusA-AR2 binding sites for αCTD, NusG-NTD and SuhB not only overlap, but all these factors are able to remove NusA-AR2 from NusA-SKK, releasing autoinhibition of NusA^18,39,41^. Thus, we explored if λQ has the same ability employing NMR-based displacement experiments. In control experiments we titrated ^2^H,^15^N-labeled NusA-SKK with NusA-AR2 and *vice versa* and recorded 1D and 2D [^1^H,^15^N] correlation spectra after each titration step to determine the chemical shift perturbations caused by NusA-AR2:NusA-SKK complex formation on both sides (Fig. 5a,b and Supplementary Fig. 11a,b). For both titrations normalized chemical shift perturbations were plotted against the amino acid sequence of the labeled protein (Fig. 5a,b) and mapped on the 3D structures (Fig. 5c). The identified binding surfaces were in agreement with previous data^39^, i.e. the C-terminal part of NusA-AR2 is affected as well as the KH1 domain of NusA-SKK. Based on the normalized changes of the chemical shifts we estimated the affinity of the NusA-SKK:NusA-AR2 interaction to be <341 µM (Supplementary Fig. 11c). In an alternative approach we determined the *K*_D_ value by fluorescence anisotropy measurement employing the NusA-AR2^D443C^ variant, yielding a slightly lower affinity (279 ± 17 µM; Supplementary Fig. 11d), similar to the one of the NusA-AR2^D443C^:λQ interaction. As no structure of autoinhibited NusA is available we performed NMR-guided docking using the results of the HSQC titrations and without allowing for conformational changes to obtain a model of the NusA-SKK:NusA-AR2 complex (Fig. 5c). NusA-AR2 packs tightly against the KH1 domain via its C-terminal helix with an interaction surface of 1230 Å^2^, blocking the RNA binding site. Addition of λQ to a preformed ^2^H,^15^N-NusA-SKK:NusA-AR2 complex (molar ratio 1:2) led to a partial reversal of all signal shifts (Fig. 5d and Supplementary Fig. 11e). This finding is in agreement with the *K*_D_ values of the NusA-SKK:NusA-AR2 and NusA-AR2:λQ interactions and indicates that λQ binds to NusA-AR2 releasing NusA-SKK at the same time, thus being compatible with the release of NusA autoinhibition by λQ. However, one must bare in mind that isolated NusA domains were used in these experiments and that the affinity of NusA-AR2 for NusA-SKK might be higher in the full length protein due to an increased local concentration. Consequently, our results are in agreement with a λQ-induced release of autoinhibition of NusA, but not final prove. NMR-based approaches using the full length NusA protein failed due to stability issues. Repeating the competition experiment with λQ^Δ36^ confirmed that the N-terminus is not required for this function of λQ (Fig. 5e and Supplementary Fig. 11f).

## Discussion

Q-dependent AT is the second mechanism lambdoid phages use to suppress termination signals. N-mediated AT is the best studied AT mechanism by now whereas only little is known about AT relying on Q. Only recently, the structural basis for AT involving Q from bacteriophage 21 has been deciphered^12,13^. However, as mentioned before, Q proteins can be grouped into three families, Q21, Q82, and λQ, and these families show no significant amino acid sequence similarity and only very little similarity in the 3D structure (with structural information being available only for Q21 and a truncated version of λQ)^12–14^. Consequently, the molecular mechanisms they use to achieve AT might be completely different, despite the fact that all Q proteins seem to bind to or in the vicinity of the βFTH in order to affect pausing and termination^12,13,43,45^. One striking difference between Q21 and λQ is for example that the latter has a long N-terminal region with unknown function that has been suggested to be unstructured. Moreover, two distinct activities have been suggested for Q82, namely antipausing and RNA occlusion, which may both play a role in Q82 function^43^. Finally, it is known that Nus factors are involved in other AT mechanisms such as N-mediated and ribosomal AT^6,7,15,17,18^. At least NusA has been demonstrated to influence Q function in phages λ and 82, although the dependency of Q activity on NusA differs^32,43,44,46^. Thus, we set out to identify possible interactions of λQ with Nus factors.

Using solution-state NMR spectroscopy we assigned secondary structure elements of λQ^Δ36^ and found that the solution structure is in good agreement with the crystal structure of an N-terminal truncation variant of λQ missing 38 amino acids (Fig. 1). Structural similarity to Q21 and region 4 of σ^70^ factor is limited to the helix-turn-helix (HTH) motif formed by helices α5 and α6^12,13,47,48^, which might be involved in the recognition of the QBE^14^. Moreover, we confirmed that residues 1–66 are indeed unstructured (Fig. 1). It has been proposed that at least two molecules of λQ are involved in the AT process^11^ and NMR spectroscopy and analytical SEC indicate that λQ and λQ^Δ36^ exist as monomers in solution, suggesting that the N-terminus does not induce oligomerization. This implies several scenarios: (i) dimerization/oligomerization occurs upon DNA binding or loading to the paused TEC, similar to Q21^12,13^, (ii) several Q proteins are involved in AT, but do not interact with each other, or (iii) λQ acts as monomer that contacts both suggested binding sites in the QBE on the DNA^11^.

**Figure 6:**
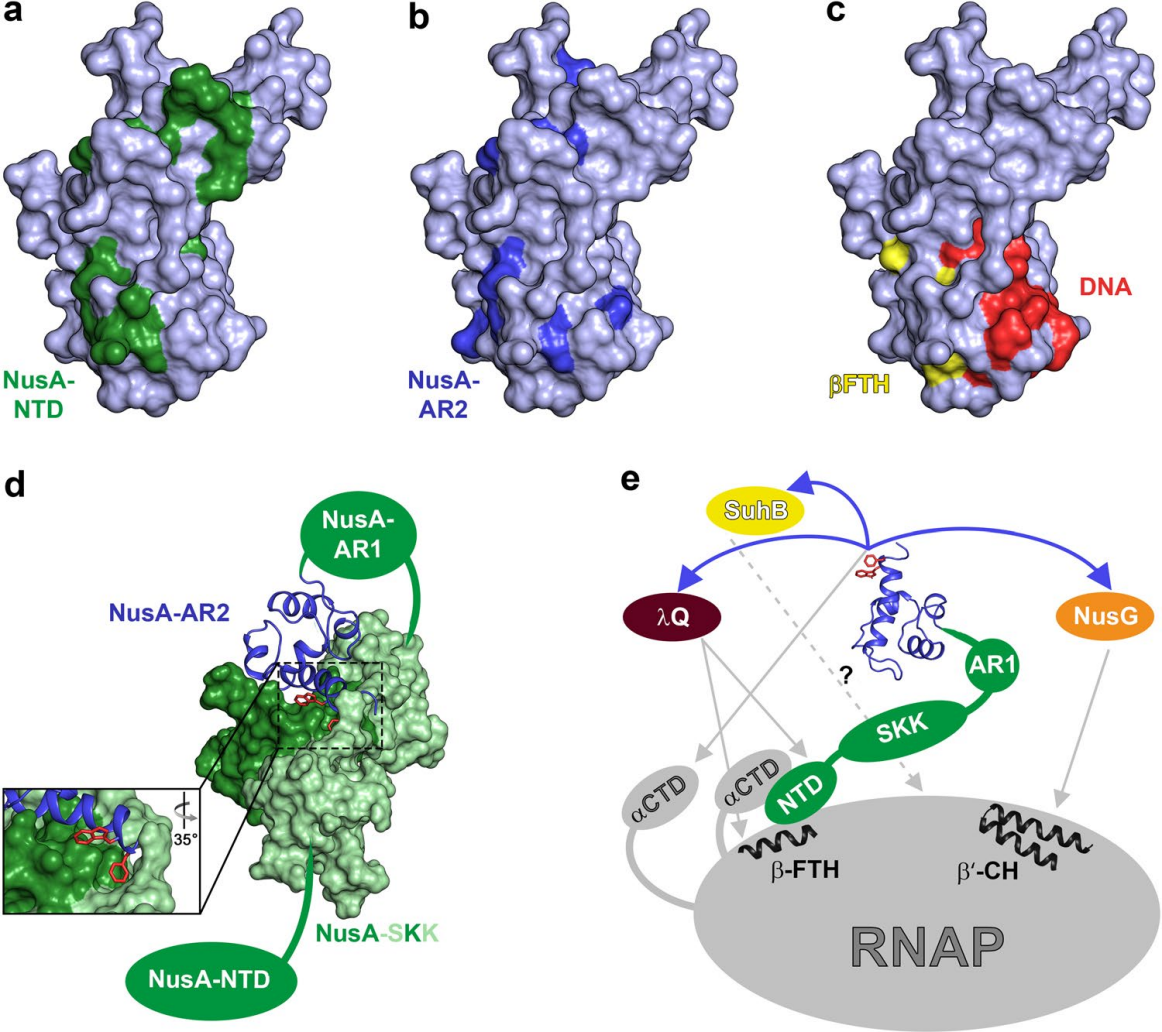
NusA functions during transcription regulation. **(a-c)** Interaction surfaces of λQ (λQ in surface representation, gray, PDB ID: 4MO1). Residues affected by NusA-NTD binding are colored in green (**a**; this study), NusA-AR2 binding in blue (**b**; this study), and βFTH and DNA binding in yellow and red, respectively (**c**; data taken from^14^). **(d)** Model of the autoinhibited state of NusA. NusA-AR2 (PDB ID: 1WCN, blue) is in ribbon, NusA-SKK (PDB ID: 5LM9, green) in surface representation, NusA-NTD and NusA-AR1 are depicted as green ellipsoids. The panel shows a magnification of the boxed region with W490 and F491 of NusA-AR2 as red sticks. **(e)** Scheme of possible roles of NusA. NusA-AR2 is shown in ribbon representation (PDB ID: 1WCN, blue) with W490 and F491 as red sticks, all other NusA domains and proteins are shown as ellipsoids and labeled. Selected RNAP binding sites are depicted in ribbon representation and labeled. Blue arrows indicate interactions of NusA-AR2 with other transcription factors, gray arrows show interactions with RNAP (the interaction of SuhB with RNAP requires further investigation and is displayed as dashed arrow). β’ clamp helices, β’CH. The PyMOL Molecular Graphics System (Version 1.7, Schrödinger, LLC.; https://pymol.org) was used for visualization of protein structures.

Q proteins bind to/near the βFTH of RNAP in order to exert their AT function^12,13,45^ and Q21 has been shown to form a nozzle at the RNA exit channel through which the nascent RNA is guided, preventing the formation of pause or termination hairpins^12,13^, i.e. AT can proceed without the need of any other factors. In contrast, Q82 is supposed to form a shield for the exiting RNA in a NusA-dependent manner^43^ and also λQ-dependent AT is stimulated by NusA^32,44,46^. Thus, we asked if λQ makes direct interactions with any of the Nus factors and we show that λQ only interacts with NusA, contacting the NTD and the AR2 domain. In neither case the N-terminus of λQ is involved in the interaction so that its function remains elusive. Interestingly, the λQ binding sites for NusA-NTD and NusA-AR2 overlap so that NusA-NTD and NusA-AR2 binding are mutually exclusive (Fig. 6a,b), as confirmed by competition experiments, suggesting similar affinities and thus distinct roles for these complexes in λQ-dependent AT. Moreover, the NusA binding sites involve (at least partially) the HTH motif (Fig. 6c) so that NusA interaction might interfere with DNA binding and might thus be relevant only once λQ is loaded to the TEC, in agreement with the fact that NusA is usually recruited after the σ factor has left the TEC^49^. If more than one λQ molecule is loaded to the TEC, interactions with NusA-NTD, NusA-AR2 and DNA would be possible simultaneously.

The NusA-AR2:λQ interaction might have various regulatory roles. It could (i) stabilize the TAC, (ii) promote the engagement of λQ with the TAC, in agreement with a previous hypothesis^32^, (iii) mediate NusA loading if NusA enters the TAC after λQ, (iv) alter the RNA binding properties of NusA by releasing autoinhibition, or (v) recruit further λQ molecules in the course of transcription, which might be necessary as the late gene region in phage λ comprises 26 kb, (vi) a combination of several of these possible functions.

During transcriptional pausing NusA-NTD interacts with βFTH and the αCTD, the latter interaction involving NusA-NTD helices α3 and parts of the preceding loop^2^. Thus the NusA-NTD:αCTD binding site overlaps with the NusA-NTD:λQ interaction surface and both interactions are mutually exclusive as demonstrated by NMR-based competition experiments (Fig. 2d,e), but λQ does not directly bind to αCTD (Supplementary Fig. 6c). Interestingly, the absence of the αCTD affects the ability of NusA to stimulate λQ-dependent AT^42^, suggesting that the NusA-NTD:αCTD and NusA-NTD:λQ interactions have relevant roles in λQ-dependent AT.

NusA-NTD, λQ, and σ region 4 bind to the βFTH, competing for this binding site^2,45,50^. Thus, we hypothesize that upon loading λQ establishes contacts with the βFTH as σ region 4 has already been disengaged from its position in the initiation complex at this stage, as shown for Q21^12,13^. Our NMR data and a mutagenesis analysis^14^ suggest that λQ, βFTH, and NusA-NTD cannot form a ternary complex as λQ binding sites for βFTH and NusA-NTD overlap, so that binding is competitive. In this case simultaneous contacts of λQ to the βFTH and NusA-NTD would only possible if more than one molecule λQ is present.

Processive λN-mediated AT involves repositioning of NusA-NTD^6,7^ and a similar mechanism has been suggested for ribosomal AT^17,18^. Although the structures of TACs and antiterminators, the time of antiterminator recruitment and the recruitment signals differ in Q-dependent, N-dependent, and ribosomal AT mechanisms^6,7,12,13,51^ the repositioning of NusA-NTD might be a general scheme in AT. Thus, based on our findings we speculate that once recruited to the βFTH λQ may alter the usual NusA:RNAP and/or NusA:RNA contacts, which would finally result in a positioning of NusA-NTD in a way that may prevent the formation of pause and termination hairpins and thus enhances elongation, rendering Q-dependent AT, just like λN-dependent AT, processive^6,7^.

NusA is a central transcriptional regulator and well conserved in bacteria. It is a multidomain protein, but only NusA from *E. coli* and some other γ-proteobacteria contains the two AR domains at the C-terminus^35^. In its isolated form, NusA is autoinhibited as NusA-AR2 binds to the KH1 domain of the SKK motif^39^, preventing RNA binding by SKK (Fig. 6d). Regulation via autoinhibition is a common scheme in the regulation of all kinds of biochemical processes. In general, autoinhibition describes the negative regulation by intramolecular interactions of different regions of the polypeptide chain, that may even be coupled to conformational changes, and that inhibit the function of at least one of the regions^52,53^. Only under certain circumstances, e.g. the binding of a specific effector, autoinhibition is released and the protein/enzyme is activated. On NusA-AR2 NusA-SKK binding involves the same region as the interaction with λQ (Figs. 3c and 5c), SuhB^18^, NusG-NTD^40^, and αCTD^39^. Moreover, all known interaction partners of NusA-AR2 are able to release the autoinhibition of NusA and may thus serve as NusA activators. This activation may occur in binary complexes or when NusA is bound to RNAP via NusA-NTD.

NusA-AR1 and NusA-AR2 have nearly identical structures with 31.5% sequence identity and contain predominantly acidic residues, resulting in a very similar electrostatic potential surface^35^. Nevertheless, each AR domain is able to recognize specific targets. NusA-AR1 specifically binds to antiterminator protein N from phage λ^35,37^, whereas several binding partners interact with NusA-AR2, e.g. λQ (Fig. 3), SuhB^17,18^, NusG-NTD^40^, and αCTD^39^. Interestingly, all these binding partners have overlapping binding sites on NusA-AR2, all involving helix α5, and all probably relying on a similar recognition mechanism based on the neighboring aromatic residues W490 and F491, located at the very C-terminus (Supplementary Fig. 12). A Leu (L414) and an Ala (A415) residue can be found at corresponding positions in NusA-AR1.

NusA is composed of several domains with the AR2 domain forming the C-terminus. As all domains are connected via flexible linkers, NusA has a high intramolecular flexibility, i.e. even when NusA is bound to RNAP via its NTD and the SKK motif to RNA during transcription, the AR2 domains can still move virtually independently. Thus, the AR2 domain may serve as flexible and versatile recruitment platform that allows the specific recruitment for various transcription factors in *E. coli* and other γ-proteobacteria (Fig. 6e), as suggested earlier^18^. Once bound to NusA-AR2 these regulators may stay at the AR2 domain or may be handed over to RNAP or other parts of the transcription machinery.

NusA has multiple, sometimes even opposing, functions, which are context- and regulator-dependent, ranging from pause-stimulation to AT. NusA is recruited early in transcription elongation^49^ and its NTD occupies the same position as region 4 of the σ factor, i.e. it binds to the βFTH, which constitutes a part of the wall of the RNA exit channel^2^. By interacting with the βFTH NusA-NTD may affect the widening of the RNA exit channel, modulating the transcription speed^2,6,7^. Additionally, NusA-NTD is contacted by one of the αCTDs^2^, suggesting that NusA-NTD would still be tethered to RNAP even if the NusA-NTD:βFTH contact was lost (e.g. if a λQ protein is bound to the βFTH). As discussed above, the AR2 domain is able to establish highly specific contacts to other transcription factors, allowing their specific recruitment to the RNAP and thus facilitate the modulation of RNAP activity. Taken together, the early recruitment of NusA, its tuneable function and its ability for the specific recruitment of various other transcription regulators not only underline the central role of NusA in transcription regulation, but imply that NusA may be regarded as auxiliary/additional RNAP subunit, similar to the σ factor, although not being encoded in the *rpo* operon.

## Methods

### Cloning and mutagenesis

The gene encoding λQ was amplified from the plasmid pUC57_lambdaq obtained from GenScript (Piscataway, NJ, USA; the gene was codon-optimized; additionally, an *Nco*I restriction site was introduced permitting the generation of a λQ deletion variant lacking 36 amino acids at the N-terminus (see below) without changing the amino acid sequence) by polymerase chain reaction using the primers Q-PciI-FW (5′-gc**acatgt**cccgcctggaatccgtggcaaaatttcac-3′; *Pci*I restriction site in bold) and Q-*Xho*I-RV (5′-g**ctcgag**tcagcgggtaacggcattcagg -3′; *Xho*I restriction site in bold; both primers were obtained from Metabion, Martinsried, Germany) and cloned into the pETGB1a expression vector (provided by Gunter Stier, EMBL Heidelberg, Germany) via *Pci*I and *Xho*I restriction sites, resulting in the recombinant plasmid pETGB1a_lambdaq. The gene coding for the Q variant lacking the 36 N-terminal residues, λQ^Δ36^, was obtained by restriction of pUC57_lambdaq with *Nco*I and *Xho*I and cloned into pETGB1a (pETGB1a_lambdaqdNΔ36). Both recombinant target proteins have a hexa-histidine tag, the B1 domain of streptococcal protein G (GB1), and a Tobacco Etch Virus (TEV) cleavage site at their N-termini.

### Gene expression and protein purification

Production of NusA was carried out as described^40^, as was production of NusA-NTD^54^, NusA-SKK^55^, NusA-AR1^37^, NusA-AR2^40^, NusA-AR2^D443C 18^, NusB^28^, NusE^Δ^:B^28^, NusG^56^, and αCTD^35^.

Expression of *λq* was carried out in *E. coli* Rosetta (DE3) plysSRARE (Novagen, Madison, USA) harboring the plasmid pETGB1a_lambdaq. Lysogeny broth (LB) medium (supplemented with 34 μg/ml chloramphenicol and 30 μg/ml kanamycin) was inoculated with an overnight preculture to an optical density at 600 nm (*OD*_600_) of 0.2 and incubated at 37 °C. When the culture reached an *OD*_600_ of 0.5 the temperature was decreased to 25 °C and overexpression was induced by addition of 0.2 mM Isopropyl β-D-1-thiogalactopyranoside (IPTG) at an *OD*_600_ of 0.7. Four hours after induction cells were harvested by centrifugation (6,000 × *g*, 10 min, 4 °C), resuspended in buffer Q-A (50 mM Tris(hydroxymethyl)aminomethane (Tris)/HCl, pH 7.4, 250 mM NaCl, 5 mM Dithiothreitol (DTT)), and lyzed using a microfluidizer (Microfluidics, Newton, USA). The lysate was cleared by centrifugation (75,000 × *g*, 30 min, 4 °C) and the crude extract was filtrated (0.45 µm filter) before being applied to a 5 ml HisTrap HP column (GE Healthcare, Chalfont St Giles, UK) loaded with Zn^2+^ instead of Ni^2+^-Ions. Upon washing with buffer Q-A elution was carried out via a step gradient with increasing imidazole concentrations (10 mM–500 mM in buffer Q-A). Fractions that contained His_6_-Gb1-λQ were combined and dialyzed against buffer Q-A (molecular weight cut-off (MWCO) 3,500 Da) at 4 °C overnight in the presence of TEV protease. The dialysate was loaded on a 5 ml HisTrap HP column (loaded with Zn^2+^) coupled to a 5 ml Heparin HP column (GE Healthcare, Munich, Germany) and the columns were washed with buffer Q-A. Subsequently, the HisTrap HP chelating column was removed and the Heparin HP column was eluted using a constant gradient from 250 mM to 1 M NaCl in buffer Q-A. Fractions that contained λQ protein were combined and dialyzed against buffer Q-B (50 mM 3-(*N*-morpholino)propanesulfonic acid (MOPS) buffer, pH 6.5, 300 mM NaCl, 150 mM D-Glucose, 5 mM DTT; MWCO 3,500 Da) at 4 °C. The protein was polished by a gel filtration step using a Superdex75 10/600 column (GE Healthcare, Munich, Germany) and buffer Q-B. Fractions containing pure λQ were concentrated by ultrafiltration (MWCO 3,000 Da), shock frozen in liquid nitrogen, and stored at −80 °C. The production of λQ^Δ36^ was carried out analogously.

### Analytical gel filtration of λQ and λQΔ^36^

Analytical gel filtration was carried out using a Superdex 75 10/300 GL column (GE Healthcare, Munich, Germany; 20 mM Tris/HCl (pH 7.5), 150 mM NaCl, 5% (v/v) glycerol). In order to estimate the molecular weights of λQ and λQ^Δ36^ a standard curve was generated using aprotinin (6.5 kDa; Sigma-Aldrich, Darmstadt, Germany), ribonuclease (13.7 kDa; GE Healthcare, Munich, Germany), carbonic anhydrase (29.0 kDa; GE Healthcare, Munich, Germany), ovalbumin (43 kDa; GE Healthcare, Munich, Germany), and albumin (66.0 kDa; Sigma-Aldrich, Darmstadt, Germany). 250 µg protein were applied per run.

### Quality control of recombinant proteins

The quality of proteins used in this study was assessed based on the guidelines established by ARBRE-MOBIEU and P4EU (https://arbre-mobieu.eu/guidelines-on-protein-quality-control/). Sodium dodecyl sulfate polyacrylamide gel electrophoresis (SDS-PAGE) was used to check the purity of proteins. UV/visible spectra from 220–340 nm were recorded on a Nanodrop ND-100 spectrometer (PEQLAB, Erlangen, Germany) to ensure the absence of nucleic acids and aggregation. In order to determine concentrations the absorbance at 280 nm was measured in a 10 mm quartz cuvette (Hellma, Müllheim, Germany) on a Biospectrometer basic (Eppendorf, Hamburg, Germany). Identity was confirmed by peptide mass fingerprinting (Department of Biochemistry, University of Bayreuth, Germany) and homogeneity was ensured by analytical gel filtration using a Superdex 75 or a Superdex 200 10/300 GL column (GE Healthcare, Munich, Germany). Circular dichroism (CD) spectroscopy (1 mm quartz cuvette; J-1100, JASCO, Pfungstadt, Germany) was performed to assess the folding state (the folding state of unlabeled λQ and λQ^Δ36^ was additionally checked by recording one dimensional ^1^H-NMR spectra).

### Isotopic labeling of proteins

Isotopic labeling was essentially based on published protocols^54^. To produce uniformly ^15^N- or ^15^N,^13^C-labeled proteins *E. coli* cells were grown in minimal medium M9^57,58^ containing (^15^NH_4_)_2_SO_4_ (CortecNet, Voisins-Le-Bretonneux, France) or (^15^NH_4_)_2_SO_4_ and ^13^C-glucose (Campro Scientific, Berlin, Germany) as sole source for nitrogen or carbon, respectively. For expression of *λq* and *λq*^*Δ*36^ Fe(III) citrate and the trace element solution were omitted, but ZnCl_2_ was added to a final concentration of 4 ^mg^/_ml_. Deuterated proteins were produced by growing *E. coli* cells in M9 medium^57,58^ which was prepared with stepwise increasing amounts of D_2_O (25% (v/v), 50% (v/v), 99.9% (v/v) D_2_O; Eurisotop, Saint-Aubin, France). Expression and purification protocols were the same as for proteins produced in LB medium.

### NMR spectroscopy

NMR experiments were performed at 298 K on Bruker *Avance* 700 MHz, Bruker *Ascend Aeon* 900 MHz, and Bruker *Ascend Aeon* 1 000 MHz spectrometers, all being equipped with cryogenically cooled inverse triple resonance probes. The experimental setup and data analysis was done essentially as described^18^. Samples contained 10% (v/v) D_2_O for locking and were in 3 mm tubes with an initial volume of 250 µl, if not stated otherwise. In-house routines were used for data conversion and processing, MatLab (The MathWorks, Inc., Version 7.1.0.183) was used for visualization and analysis of one-dimensional (1D) spectra and NMRViewJ (One Moon Scientific, Inc., Westfield, NJ, USA) to visualize and analyze two-dimensional (2D) and three-dimensional (3D) spectra. Assignments for the backbone amide resonances of NusA-AR2^35^, NusA-AR1^59^, NusA-SKK^39^, αCTD^39^, and NusA-NTD^54^ were taken from previous studies.

For resonance assignment of the λQ^Δ36^ backbone BEST-TROSY-based triple resonance experiments^60–62^ were recorded using 5 mm tubes (500 µl sample volume) with the ^2^H,^13^C,^15^N-labeled protein (270 µM) being in 25 mM MES (pH 7.0), 100 mM NaCl, 5 mM DTT. ^15^N-longitudinal and transverse relaxation rates of λQ^Δ36^ were recorded with a ^15^N-labeled sample at 298 K and 700.2 MHz ^1^H frequency using standard methods^63^. Relaxation delays were fitted to a monoexponential decay by NMRViewJ (One Moon Scientific, Inc., Westfield, NJ, USA). The rotation correlation time was determined using the TENSOR 2 package^64^ assuming an isotropic model for molecular tumbling. Only residues located in rigid regions were used in the analysis. The error of *R*_1_ and *R*_2_ was set to 5% and 8%, respectively, according to ref. ^65^.

For interaction studies and competition experiments proteins were in 50 mM MOPS, pH 6.5, 300 mM NaCl, 150 mM D-Glucose, 5 mM DTT (exception: 5 mm tubes were used to study the interaction of NusA-SKK with NusA-AR2 with proteins being in 50 mM MOPS, pH 6.5, 100 mM NaCl, 150 mM D-Glucose, 5 mM DTT). Either [^1^H,^15^N]-HSQC or [^1^H,^15^N]-BEST-TROSY experiments were used to record [^1^H,^15^N] correlation spectra. To compare 1D spectra we normalized them by receiver gain, length of the 90° proton pulse, number of scans, and protein concentration.

[^1^H,^15^N] correlation-based titrations (either HSQC or BEST-TROSY) were analyzed quantitatively by calculating either changes in intensity or changes in chemical shifts. If chemical shift changes were in the fast regime of chemical exchange we calculated the normalized chemical shift perturbation (Δδ_norm_) according to Eq. (1).

1 $$\Delta {\delta }_{{\rm{norm}}}=\sqrt{{(\Delta {\delta }^{1}H)}^{2}+{[0.1\cdot (\Delta {\delta }^{15}N)]}^{2}}$$

with Δδ being the resonance frequency difference in ppm.

Plotting of Δδ_norm_ against the amino acid position of the labeled protein and introduction of thresholds at 0.04 ppm, 0.08 ppm, and 0.12 ppm allowed the identification of slightly, moderately, and strongly affected residues. In order to determine dissociation constants (*K*_D_) from these titrations we analyzed the normalized chemical shift changes (in Hz) and fitted a two-state model (Eq. 2) to the chemical shift change of amide protons showing fast exchange in the chemical shift timescale.

2 $$\varDelta \nu =\varDelta {\nu }_{End}\cdot \frac{{[P]}_{0}\cdot r+{[P]}_{0}+{K}_{D}-\sqrt{{({K}_{D}+{[P]}_{0}+{[P]}_{0}\cdot r)}^{2}-4\cdot {({[P]}_{0})}^{2}\cdot r}}{2\cdot {[P]}_{0}}$$

with *Δν* being the normalized resonance frequency difference (Hz), *Δν*_End_ the normalized resonance frequency difference between free and fully bound protein (Hz)*, r* the ratio of unlabeled to labeled protein, and *[P]*_0_ the total concentration of ^15^N-labeled protein (the decrease of *[P]*_0_ due to dilution was taken into account during fitting). Fitting was done using MatLab (The MathWorks, Inc., Version 7.1.0.183) with *K*_*D*_ and *Δν*_*End*_ being fitting parameters.

If the system was in slow or intermediate chemical exchange the signal intensities were analyzed quantitatively as described^66^. In brief, signal intensities were normalized by receiver gain, length of the 90° proton pulse, number of scans, and protein concentration. In order to eliminate an intensity decrease due to slight precipitation signals within one spectrum were normalized to the most intense signal. Subsequently, we calculated the relative signal intensity in each titration step, i.e. the ratio of the remaining, normalized signal intensity of the spectrum of the respective titration step to the normalized signal intensity of the spectrum of the free, labeled protein. The error was calculated based on the standard deviation of the noise level applying error propagation. Then, we calculated the mean value of all relative signal intensities in each titration step and residues with relative signal intensities below thresholds at 1 and 1.5 σ of the mean value were classified as moderately or strongly affected, respectively.

### Fluorescence anisotropy measurements

Fluorescence anisotropy measurements were performed as described^18^. Site-specific labeling of NusA-AR2^D443C^ with fluorescein-5-maleimide (ThermoFisher Scientific, Waltham, USA) was done according to the manufacturer’s protocol, i.e. after incubation of 25 µM of NusA-AR2^D443C^ with 750 µM fluorescein-5-maleimide in labeling buffer (20 mM Na phosphate, pH 7.0, 150 mM NaCl) at 4 °C overnight the solution was loaded on a PD MiniTrap Sephadex G-25 gravity column (GE Healthcare, Munich, Germany) equilibrated with fluorescence buffer (50 mM Na-P, pH 6.5, 100 mM NaCl, 150 mM glucose, 5 mM DTT, 0.05% (v/v) Tween). Elution was carried out with fluorescence buffer. The protein concentration and the degree of labeling were determined by UV/vis spectroscopy on a Nanodrop ND-1000 spectrometer (PEQLAB, Erlangen, Germany) according to the manufacturer’s protocol.

For each titration step individual 100 µl samples were prepared with each sample containing 25 nM labeled NusA-AR2^D443C^ and increasing concentrations of unlabeled protein. All proteins were in fluorescence buffer and measurements were done in black, sterile 96-well microtiter plates (Brand, Wertheim, Germany) at 25 °C in a Synergy 2 microplate reader (BioTek, Winooski, USA). Four independent replicates were prepared per titration step and the anisotropy values were averaged. Finally, the mean anisotropy values were plotted against the titrant concentration and anisotropy data was fitted to a two-state binding model (Eq. 3) using GraFit 5.0 (Erithacus Software; http://www.erithacus.com/grafit/index.html).

3 $$A=\frac{{A}_{S}+[{\rm{complex}}]/{[S]}_{0}\cdot (R\cdot {A}_{{\rm{complex}}}-{A}_{S})}{1-[{\rm{complex}}]/{[S]}_{0}+R\cdot [{\rm{complex}}]/{[S]}_{0}}$$

with

4 $$[{\rm{complex}}]=\frac{{K}_{D}+{[P]}_{0}+{[S]}_{0}-\sqrt{{({K}_{D}+{[P]}_{0}+{[S]}_{0})}^{2}-4\cdot [S]\cdot {[P]}_{0}}}{2}$$

where *A* is the measured anisotropy, *A*_S_ the anisotropy of labeled NusA-AR2^D443C^, *A*_complex_ the anisotropy of the complex, [complex] the concentration of the complex, [S]_0_ and [P]_0_ the total concentrations of labeled NusA-AR2^D443C^ and the titrant, respectively, *K*_D_ the dissociation constant, and *R* the ratio of the fluorescence intensities of fully bound and free substrate at 520 nm.

### Docking

The complexes λQ:NusA-NTD, λQ:NusA-AR2, and NusA-AR2:NusA-SKK were modeled with the HADDOCK 2.2 server (https://haddock.science.uu.nl/services/HADDOCK2.2/)^67^ using H-N correlation data from NMR titrations as restraints (Supplementary Table 1). The size of interaction interfaces was calculated via the “Protein interfaces, surfaces and assemblies” service PISA at the European Bioinformatics Institute (http://www.ebi.ac.uk/pdbe/prot_int/pstart.html^68^.

### Visualization of protein structures

The PyMOL Molecular Graphics System (Version 1.7, Schrödinger, LLC.; https://pymol.org) was used for graphical representations of protein structures.

## Data Availability

The chemical shift assignment of λQ^Δ36^ were deposited in the Biological Magnetic Resonance Data Bank under the accession code 28043. We generated models of the λQ:NusA-NTD, the λQ:NusA-AR2, and the NusA-SKK:NusA-AR2 complex. Coordinates for λQ, NusA-NTD, NusA-SKK, and NusA-AR2 are available in the Protein Data Bank (PDB; 4MO1, 2KWP, 5LM9, 1WCN), the coordinates of the best complex models are provided as Supplementary data. Other data and materials are available from the corresponding author upon reasonable request.

## Supplementary information

Supplementary Information.

Supplementary Information2.

Supplementary Information3.

Supplementary Information4.
