# Supplementary material for: NusA directly interacts with antitermination factor Q from phage λ
Source: Sci Rep. 2020 Apr 20;10:6607. doi: 10.1038/s41598-020-63523-5 (PMC7171158; doi:10.1038/s41598-020-63523-5)
Supplement: Supplementary file 1 — Supplementary Information. [file 41598_2020_63523_MOESM1_ESM.pdf]

Supplementary Information

NusA directly interacts with antitermination factor Q from phage λ

Benjamin R. Dudenhoeffer[1], Jan Borggräfe[1,2], Kristian Schweimer[1], Stefan H. Knauer[1,*]

[1]Biopolymers, University of Bayreuth, Universitätsstraße 30, 95447 Bayreuth, Germany

[2]Current address:

Institute of Physical Biology, Heinrich-Heine-University, Univeristätsstraße 1, 40225 Düsseldorf, Germany

Institute of Complex Systems, Forschungszentrum Jülich, Wilhelm-Johnen-Straße, 52428 Jülich, Germany

*Correspondence to: stefan.knauer@uni-bayreuth.de

Supplementary Table 1: Structures and active residues used in docking to model the complexes λQ:NusA-NTD, λQ:NusA-AR2, and NusA-AR2:NusA-SKK.

complex	Input structures	Active residues
λQ:NusA-NTD	λQ: 4MOI, chain A<br><br>NusA-NTD: 2KWP, model 1	λQ: 84, 86, 102, 111, 112, 117, 119, 121, 124, 127, 146, 147, 150, 158, 160, 161, 162, 163, 168, 183, 185, 186<br><br>NusA-NTD: 27, 34, 37, 38, 39, 44, 57, 61, 73, 75, 77, 80, 83, 84, 87, 95
λQ:NusA-AR2	λQ: 4MOI, chain A<br><br>NusA-AR2: 1WCN, model 1	λQ: 71, 72, 75, 95, 96, 102, 103, 111, 112, 117, 125, 149, 161, 163, 177, 180, 183, 200<br><br>NusA-AR2: 462, 463, 476, 483, 487, 489, 490, 491, 492, 494, 495
NusA-AR2:NusA-SKK	NusA-AR2: 1WCN, model 1<br><br>NusA-SKK: 5LM9 (the structure also includes NusA-AR1)	NusA-AR2: 461, 463, 464, 465, 466, 476, 480, 482, 483, 484, 486, 490, 491, 492, 493<br><br>NusA-SKK: 165, 168, 170, 171, 173, 181, 212, 229, 234, 235, 236, 249, 252, 253, 254, 258, 259, 260, 261, 262, 263, 264, 266, 267, 268, 270, 271, 273, 275, 276, 288, 289, 317, 318, 319, 326, 327, 342, 346

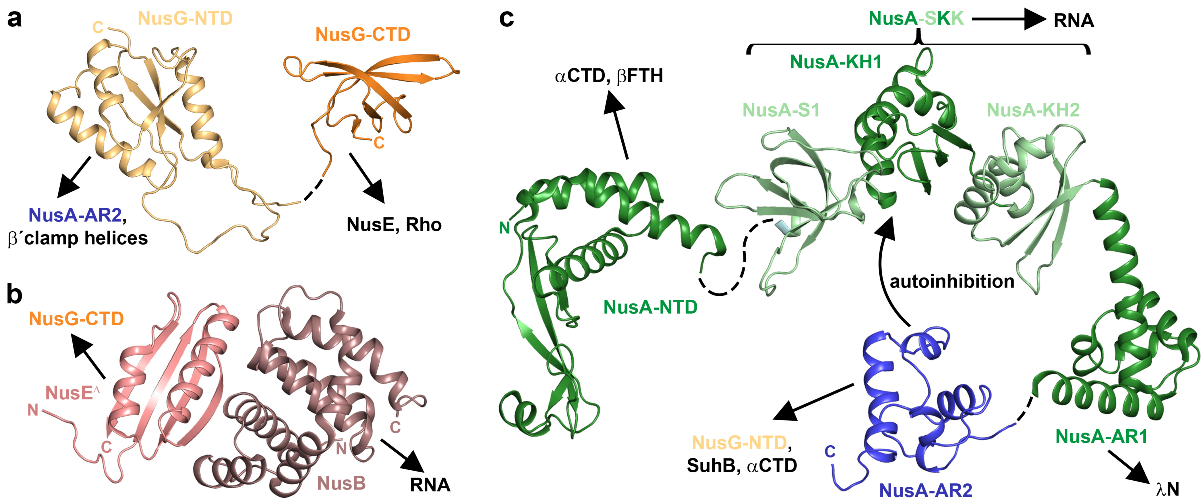

Supplementary Figure 1: Structures of Nus factors. The structures of NusG **(a)**, the NusB:NusE$^{\Delta}$ complex **(b)**, and NusA **(c)** are shown in ribbon representation, domains are color-coded, and flexible linkers are depicted as dashed lines. Termini are labeled and interaction partners are indicated. PDB IDs: NusG-NTD, 2K06; NusG-CTD, 2JVV; NusB: NusE$^{\Delta}$, 3D3B; NusA-NTD, 2KWP; NusA-SKK-AR1, 5LM9; NusA-AR2, 1WCN. The PyMOL Molecular Graphics System (Version 1.7, Schrödinger, LLC.; https://pymol.org) was used for visualization.

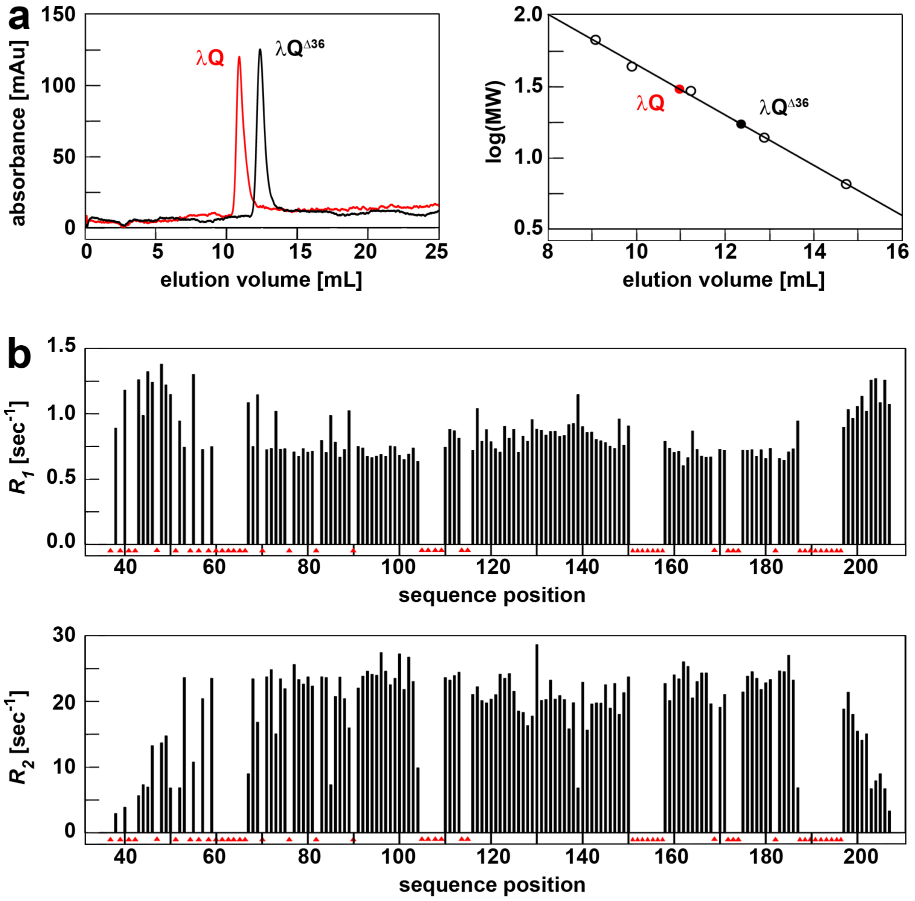

Supplementary Figure 2: Characterization of λQ and λQ$^{\Delta36}$. (a) Analytical gel filtration using a Superdex 75 10/300 GL column. (Left) Chromatograms of λQ and λQ$^{\Delta36}$. (Right) Standard curve generated with aprotinin, ribonuclease, carbonic anhydrase, ovalbumin, and albumin (empty black circles). The molecular weights of λQ (black filled circle) and λQ$^{\Delta36}$ (red filled circle) were determined as 30 kDa and 17 kDa, respectively. **(b)** Determination of relaxation rates R_1 (top) and R_2 (bottom) of ^{15}N-λQ$^{\Delta36}$. Red triangles mark residues for which no relaxation rates could be determined.

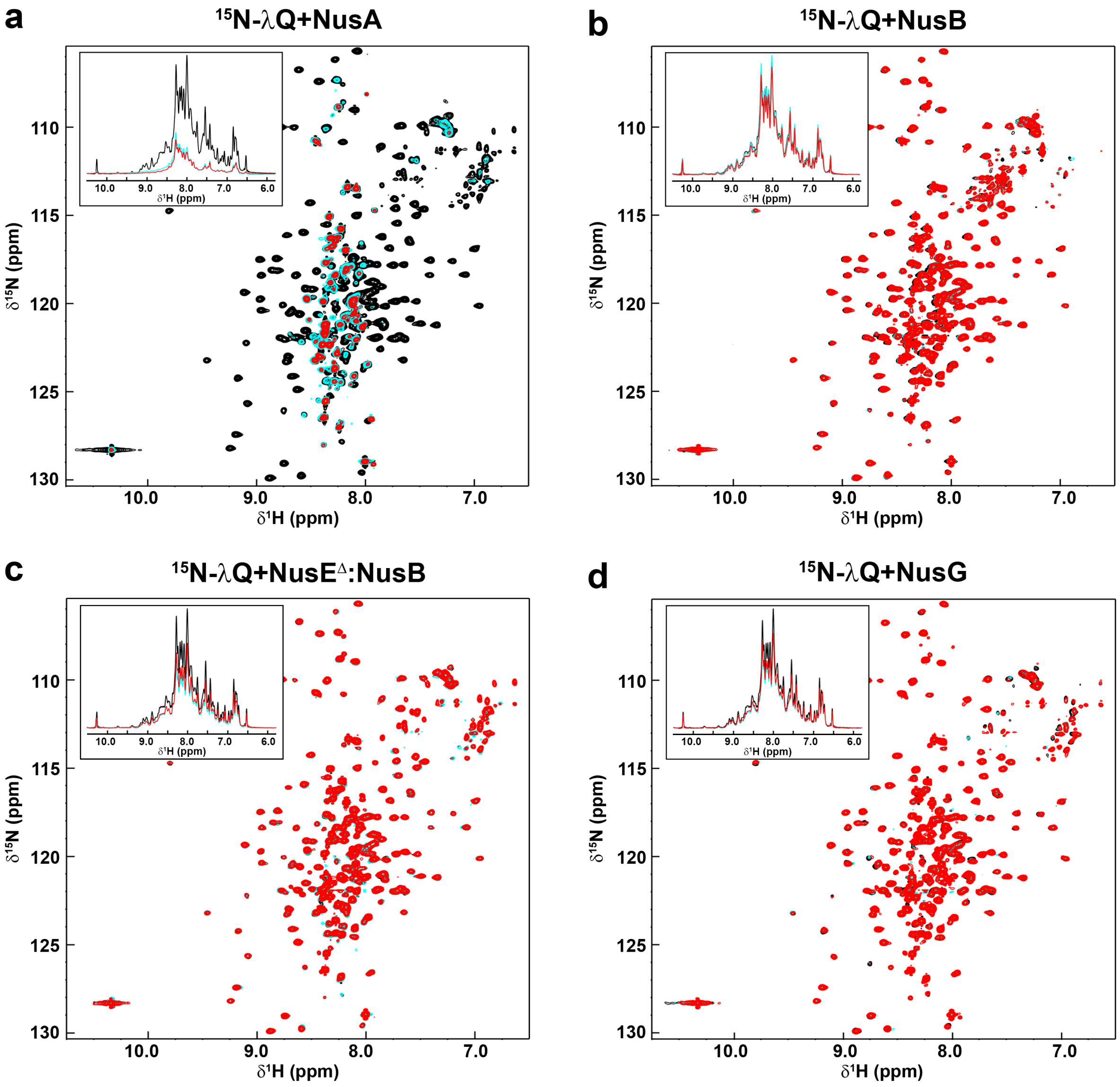

Supplementary Figure 3: λQ interacts with NusA, but not with NusB, NusE$^{\Delta}$, or NusG. 2D [^{1}H,^{15}N]-BEST-TROSY spectra of 125 µM ^{15}N-λQ in the absence (black) and presence (molar ratio 1:1, cyan; 1:2, red) of NusA **(a),** NusB **(b)**, NusB:NusE$^{\Delta}$ **(c)**, and NusG **(d)**. Corresponding 1D [^{1}H,^{15}N]-HSQC spectra are shown as insets.

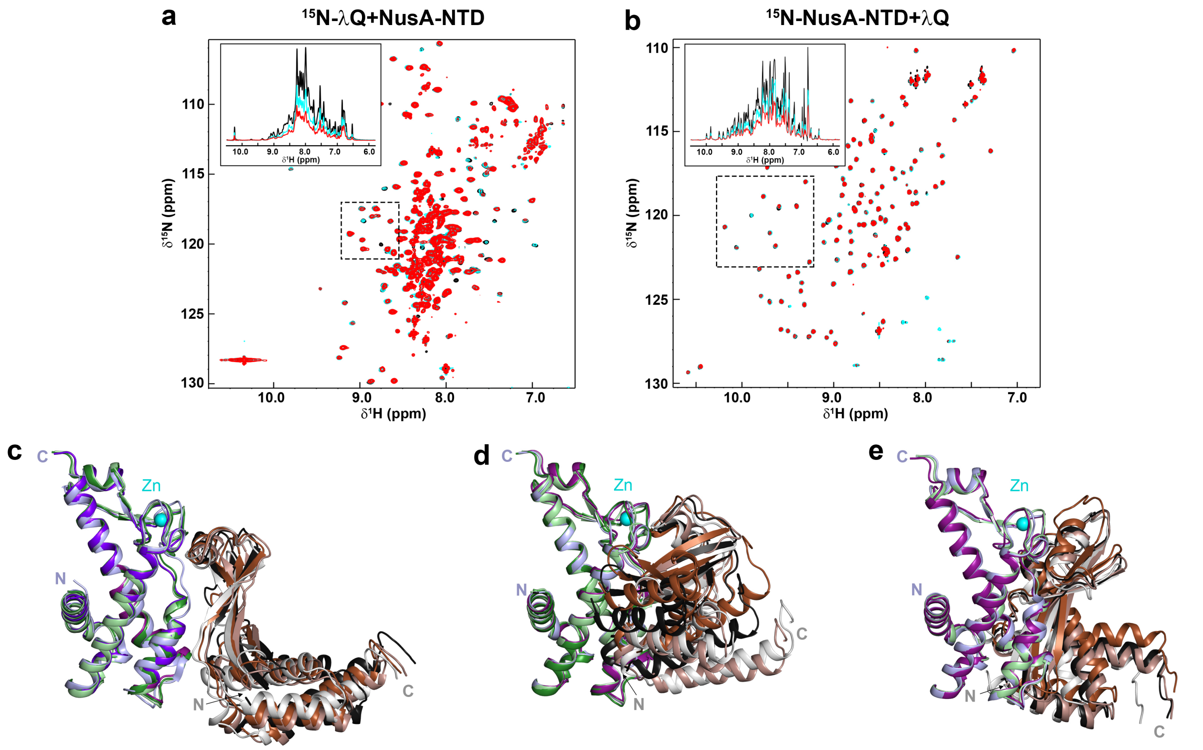

Supplementary Figure 4: λQ directly interacts with NusA-NTD. **(a)** 2D [^{1}H,^{15}N]-BEST-TROSY spectra of the titration of 250 µM ^{15}N-λQ with NusA-NTD (molar ratios: 1:0, black; 1:1, cyan; 1:2 red). The dashed box indicates the section shown in Fig. 2a. Corresponding 1D [^{1}H,^{15}N]-HSQC spectra are shown as inset. **(b)** 1D and 2D [^{1}H,^{15}N]-HSQC spectra of the titration of 175 µM ^{15}N-NusA-NTD with λQ (molar ratios: 1:0, black; 1:1, cyan; 1:2). The dashed box in **(b)** indicate the sections shown in Fig. 2b. **(c-e)** Models of the λQ:NusA-NTD complex. The models were generated with by docking (HADDOCK 2.2 server (https://haddock.science.uu.nl/services/HADDOCK2.2/) using the affected residues as restraints. The three clusters with the lowest Z and HADDOCK scores are shown (increasing values from **(c)** to **(e)**) with each cluster having three superimposed structures. The proteins are depicted in ribbon representation, the Zn^{2+} ion is shown as cyan sphere. PDB IDs: λQ, 4MOI; NusA-NTD, 2KWP. The PyMOL Molecular Graphics System (Version 1.7, Schrödinger, LLC.; https://pymol.org) was used for visualization.

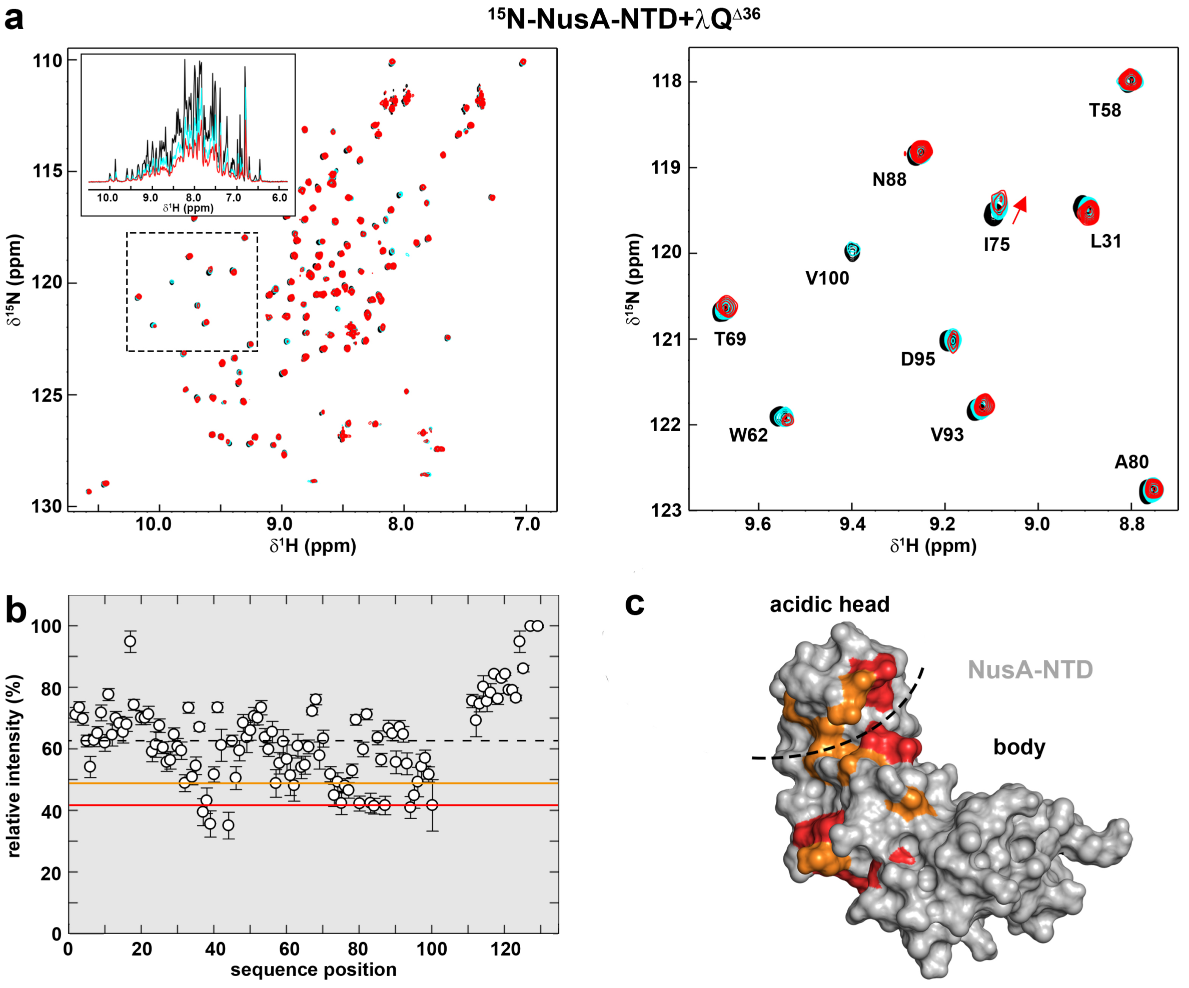

Supplementary Figure S5: λQ$^{\Delta36}$ directly interacts with NusA-NTD. **(a)** 1D and 2D [^{1}H,^{15}N]-HSQC spectra of the titration of 175 µM ^{15}N-NusA-NTD with λQ$^{\Delta36}$ (molar ratios: 1:0, black; 1:1, cyan; 1:2). The dashed box in **(a)** indicate the section marks the section shown on the right. **(b)** Relative intensity of NusA-NTD signals in the presence of one equivalent λQ$^{\Delta36}$. Orange and red lines indicate thresholds for moderately (1.0 σ of average relative signal intensity) and strongly (1.5 σ of average relative signal intensity) affected signals, respectively. Error bars are given as black vertical lines. **(c)** Mapping of the affected residues on NusA-NTD (PDB ID: 2KWP, gray in surface representation). Moderately affected residues, orange; strongly affected residues, red. The PyMOL Molecular Graphics System (Version 1.7, Schrödinger, LLC.; https://pymol.org) was used for visualization.

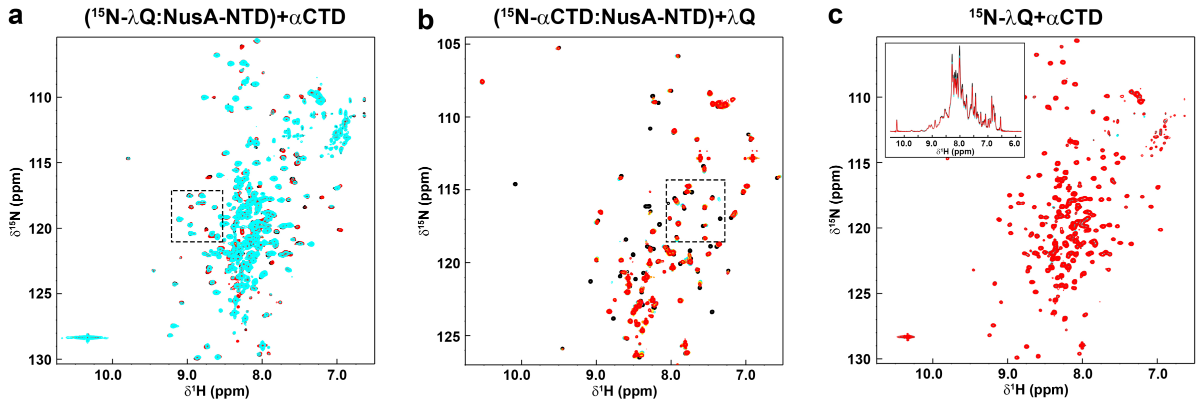

Supplementary Figure 6: The αCTD of RNAP and λQ share binding sites on NusA-NTD. (a) αCTD detaches NusA-NTD from ^{15}N-λQ. 2D [^{1}H,^{15}N]-BEST-TROSY spectra are shown. Molar ratios: ^{15}N-λQ:NusA-NTD:αCTD = 1:0:0, black; =1:2:0, cyan; 1:2:2, orange; 1:2:4, red. Initial concentration of ^{15}N-λQ: 250 μM. The dashed box indicates the section shown in Fig. 2d. **(b)** NusA-NTD is removed from ^{15}N-αCTD by λQ. 2D [^{1}H,^{15}N]-HSQC spectra are depicted. Molar ratios: ^{15}N-αCTD:NusA-NTD:λQ = 1:0:0; black; =1:2:0; cyan; 1:2:2; orange; 1:2:4, purple; 1:2:6, yellow; 1:2:10, red. Initial concentration of ^{15}N-αCTD: 250 μM. The dashed box corresponds to the section shown in Fig. 2e. **(c)** [^{1}H,^{15}N]-BEST-TROSY spectra of the titration of 125 μM ^{15}N-λQ with αCTD (molar ratios: 1:0, black; 1:1, cyan; 1:2, red). The inset shows corresponding 1D [^{1}H,^{15}N]-HSQC spectra.

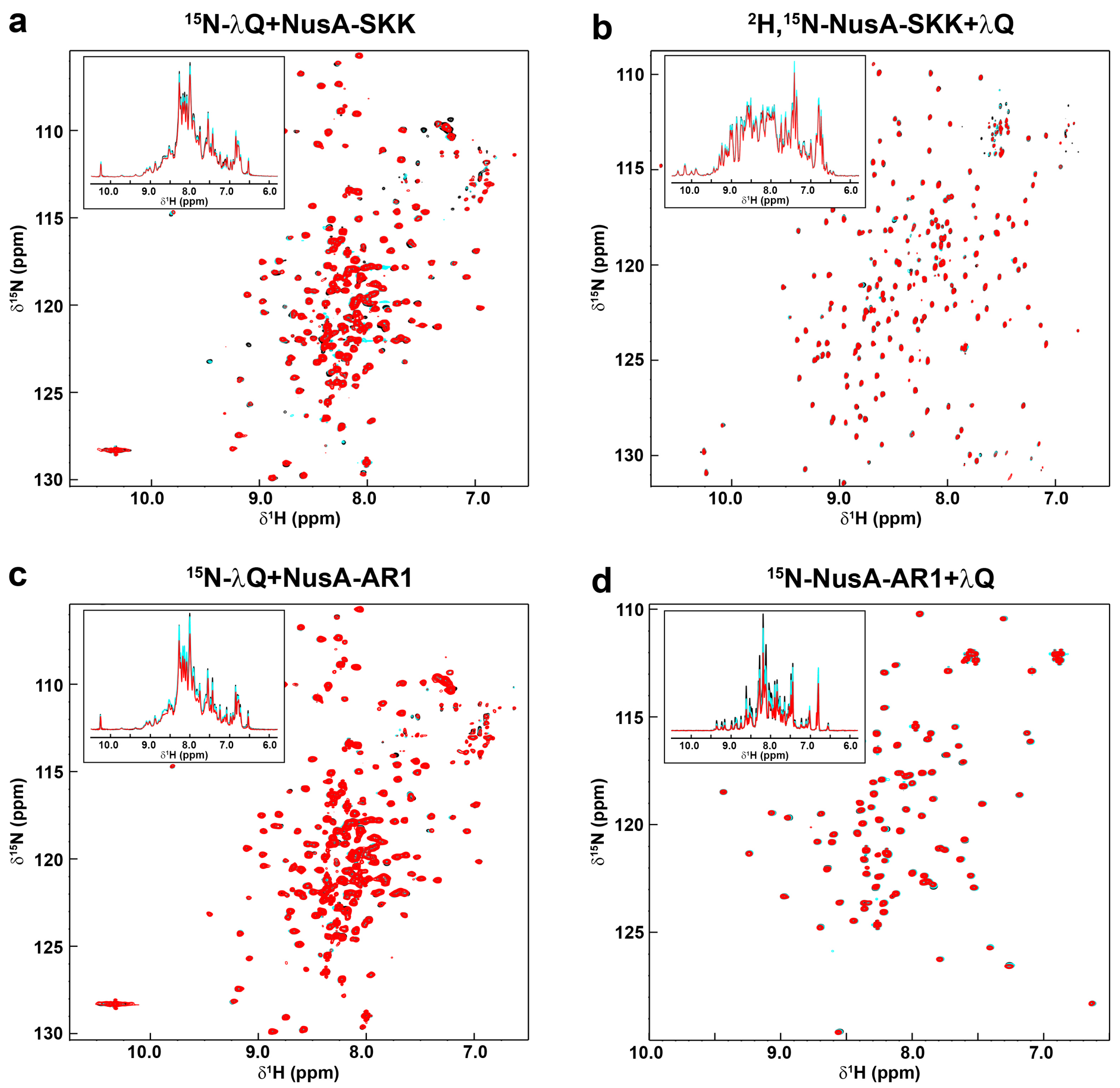

Supplementary Figure 7: λQ interacts with neither NusA-SKK nor NusA-AR1. (a) 1D [^{1}H,^{15}N]-HSQC and 2D [^{1}H,^{15}N]-BEST-TROSY spectra of the titration of 125 µM ^{15}N-λQ with NusA-SKK (molar ratios: 1:0, black; 1:1, cyan; 1:2). **(b)** 1D [^{1}H,^{15}N]-HSQC and 2D [^{1}H,^{15}N]-BEST-TROSY spectra of the titration of 125 µM ^{2}H,^{15}N-NusA-SKK with λQ (molar ratios: 1:0, black; 1:1, cyan; 1:2 red). **(c)** 1D [^{1}H,^{15}N]-HSQC and 2D [^{1}H,^{15}N]-BEST-TROSY spectra of the titration of 175 µM ^{15}N-λQ with NusA-AR1 (molar ratios: 1:0, black; 1:1, cyan; 1:2 red). **(d)** 1D and 2D [^{1}H,^{15}N]-HSQC spectra of the titration of 125 µM ^{15}N-NusA-AR1 with λQ (molar ratios: 1:0, black; 1:1, cyan; 1:2 red).

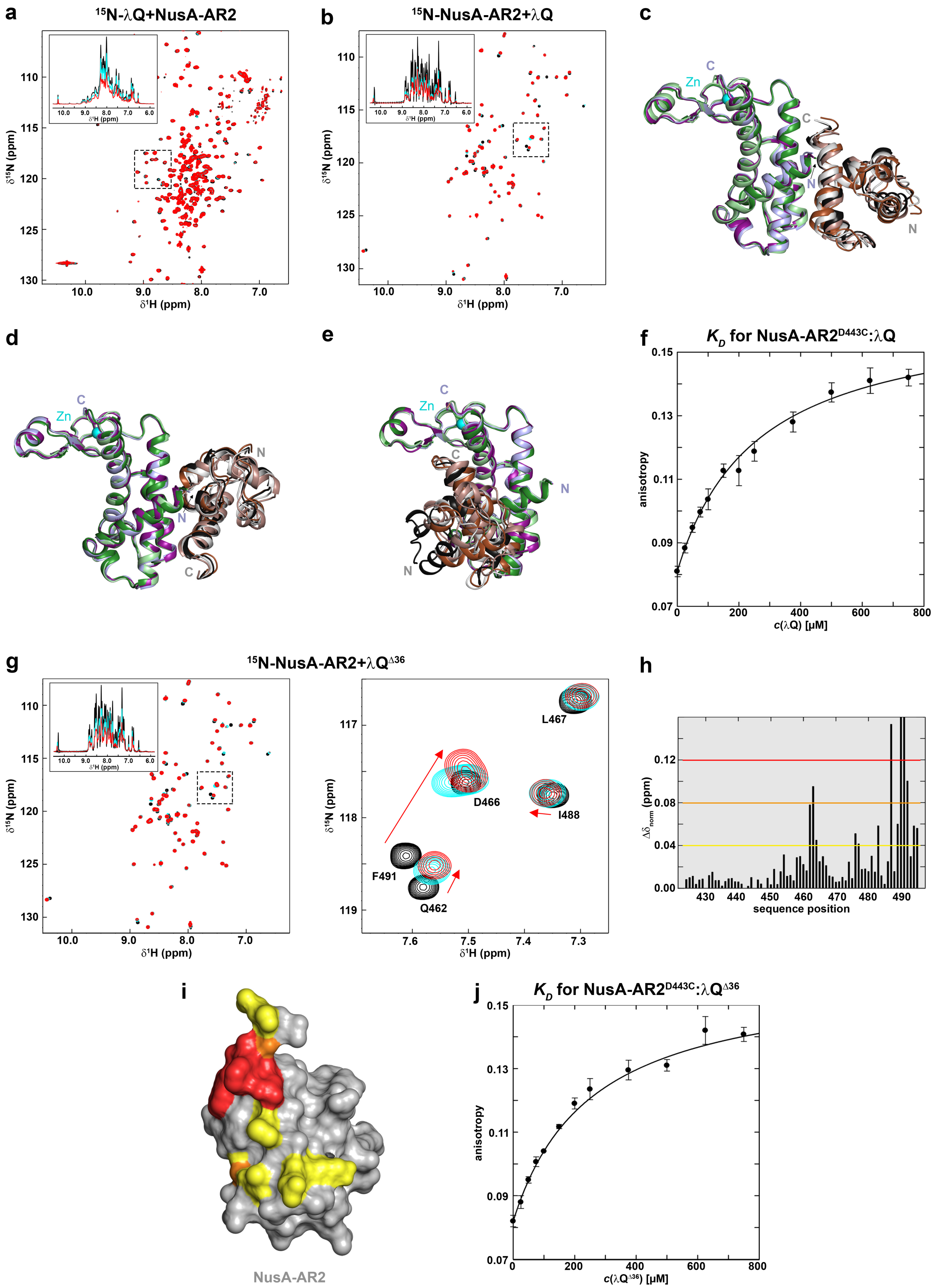

Supplementary Figure 8: λQ interacts directly with NusA-AR2. (a) 1D [^{1}H,^{15}N]-HSQC and 2D [^{1}H,^{15}N]-BEST-TROSY spectra of the titration of 250 µM ^{15}N-λQ with NusA-AR2

(molar ratios: 1:0, black; 1:1, cyan; 1:2 red). The dashed box indicates the section shown in Fig. 3a. **(b)** 1D and 2D [^{1}H,^{15}N]-HSQC spectra of the titration of 250 µM ^{15}N-NusA-AR2 with λQ (molar ratios: 1:0, black; 1:1, cyan; 1:2 red). The dashed box marks the section depicted in Fig. 3b. **(c-e)** Models of the λQ:NusA-AR2 complex. The models were generated by docking using the affected residues as restraints (the HADDOCK 2.2 server (https://haddock.science.uu.nl/services/HADDOCK2.2/). The three clusters with the lowest Z and HADDOCK scores are shown (increasing values from **(c)** to **(e)**) with each cluster comprising three superimposed structures. The proteins are depicted in ribbon representation, the Zn^{2+} ion as cyan sphere. PDB IDs: λQ, 4MOI;NusA-AR2, 1WCN. The PyMOL Molecular Graphics System (Version 1.7, Schrödinger, LLC.; https://pymol.org) was used for visualization. **(f)** Determination of the K_D of the λQ:NusA-AR2^{D443C} interaction by fluorescence anisotropy measurements. The standard deviation is shown as vertical bars. The curve represents the best fit to a two-component binding equation, yielding a K_D value of 268±17 µM. Fitting was performed with GraFit 5.0 (Erithacus Software; http://www.erithacus.com/grafit/index.html). **(g)** 1D and 2D [^{1}H,^{15}N]-HSQC spectra of the titration of 250 µM ^{15}N-NusA-AR2 with λQ$^{\Delta 36}$ (molar ratios: 1:0, black; 1:1, cyan; 1:2 red). The dashed box indicates the section shown on the right. Selected signals are assigned, arrows indicating chemical shift changes during the titration. **(h)** Normalized chemical shift changes of NusA-AR2 upon the addition of two equivalents λQ$^{\Delta 36}$. Yellow, orange, and red lines indicate thresholds for slightly (0.04 ppm ≤ $\Delta\delta_{norm}$ < 0.08 ppm), moderately (0.08 ppm ≤ $\Delta\delta_{norm}$ < 0.12 ppm) and strongly ($\Delta\delta_{norm}$ ≥ 0.12 ppm) affected signals, respectively. **(i)** Mapping of the affected residues from **(h)** on the structure of NusA-AR2 (PDB ID: 1WCN, gray, in surface representation). Slightly affected residues, yellow; Moderately affected residues, orange; strongly affected residues, red. The PyMOL Molecular Graphics System (Version 1.7, Schrödinger, LLC.; https://pymol.org) was used for visualization. **(j)** Determination of the affinity of the λQ$^{\Delta 36}$:NusA-AR2^{D443C} interaction by fluorescence

anisotropy measurements. Representation as in **(f)**. The curve represents the best fit to a two-component binding equation, yielding a K_D value of 268±17 μM. Fitting was performed with GraFit 5.0 (Erithacus Software; http://www.erithacus.com/grafit/index.html).

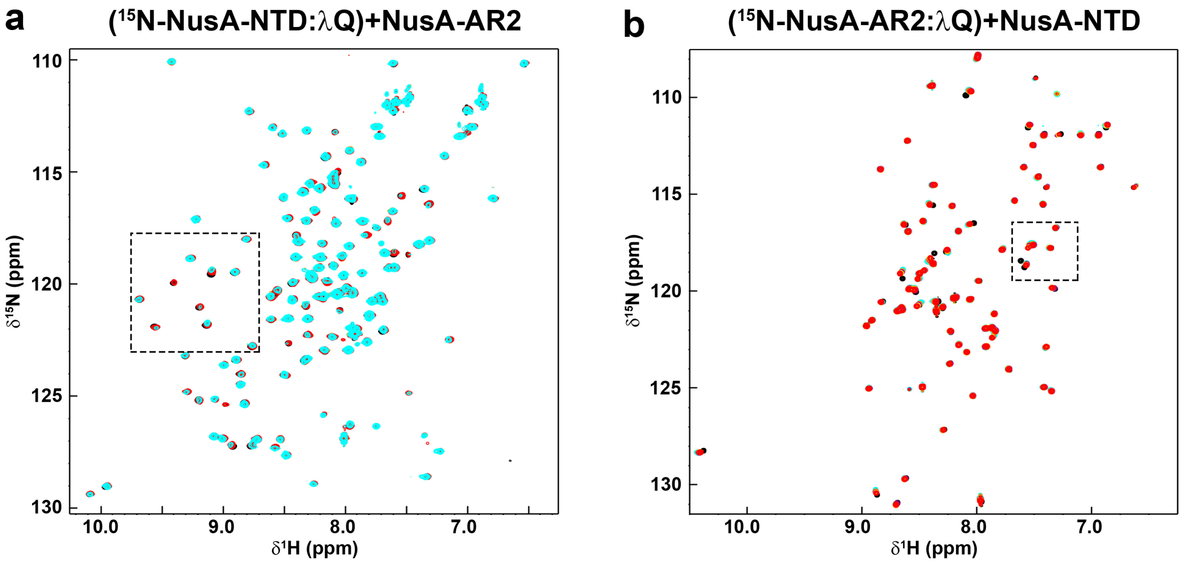

Supplementary Figure 9: NMR-based competition experiments of λQ, NusA-NTD and NusA-AR2. (a) NusA-AR2 detaches λQ from ^{15}N-NusA-NTD. 2D [^{1}H,^{15}N]-HSQC spectra are shown, dashed boxes indicate the sections shown in Fig. 3d. Molar ratios: ^{15}N-NusA-NTD:λQ:NusA-AR2 = 1:0:0, black; =1:2:0, cyan; 1:2:2, orange; 1:2:4, red. Initial concentration of ^{15}N-NusA-NTD: 250 µM. **(b)** λQ is removed from ^{15}N-NusA-AR2 by NusA-NTD. 2D [^{1}H,^{15}N]-HSQC spectra are depicted with the dashed box marking the region shown in Fig. 3e. Molar ratios: ^{15}N-NusA-AR2:λQ:NusA-NTD = 1:0:0; black; =1:2:0; cyan; 1:2:2; orange; 1:2:4, purple; 1:2:6, yellow; 1:2:10, red. Initial concentration of ^{15}N-NusA-AR2: 250 µM.

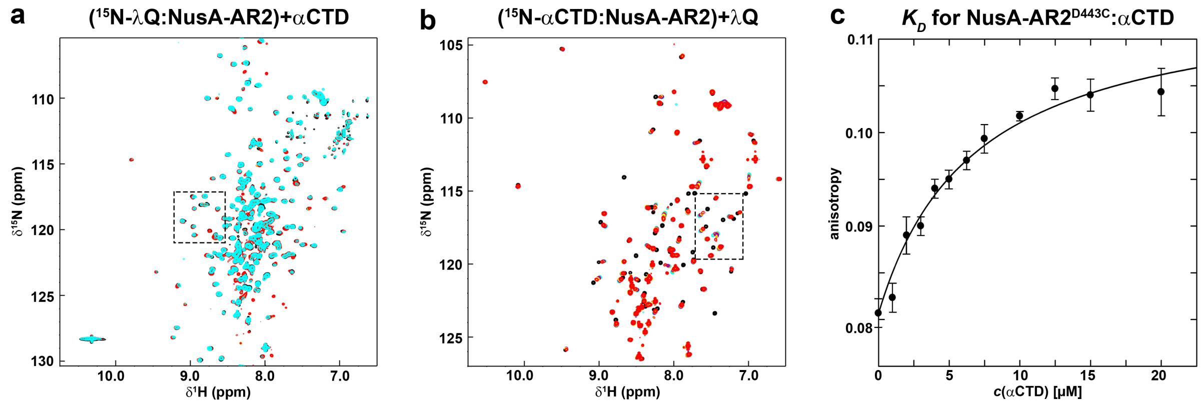

Supplementary Figure 10: The αCTD and λQ share binding sites on NusA-AR2. (a) Removal of NusA-AR2 from ^{15}N-λQ by αCTD. 2D [^{1}H,^{15}N]-BEST-TROSY spectra are shown, the dashed box indicates the region shown in Fig. 4a. Molar ratios: ^{15}N-λQ:NusA-AR2:αCTD = 1:0:0, black; =1:2:0, cyan; 1:2:2, orange; 1:2:4, red. Initial concentration of ^{15}N-λQ: 250 µM. **(b)** λQ detaches NusA-AR2 from ^{15}N-αCTD. 2D [^{1}H,^{15}N]-HSQC spectra are depicted, the dashed box marks the section magnified in Fig. 4b. Molar ratios: ^{15}N-αCTD:NusA-AR2:λQ = 1:0:0; black; =1:2:0; cyan; 1:2:2; orange; 1:2:4, purple; 1:2:6, yellow; 1:2:10, red. Initial concentration of ^{15}N-αCTD: 250 µM. **(c)** Determination of the affinity of the αCTD:NusA-AR2^{D443C} interaction by fluorescence anisotropy measurements. The standard deviation is shown as bars. The curve represents the best fit to a two-component binding equation, yielding a K_D value of 8±1 µM. Fitting was performed with GraFit 5.0 (Erithacus Software; http://www.erithacus.com/grafit/index.html).

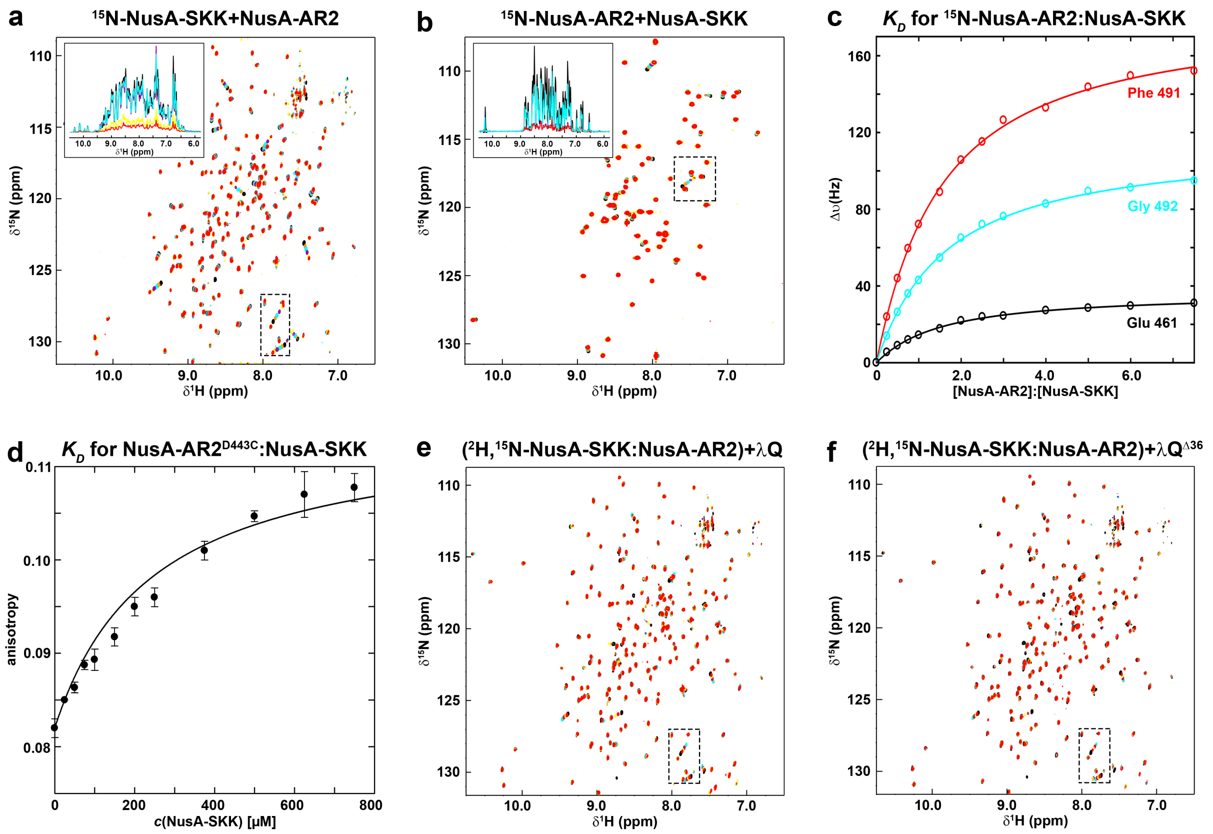

Supplementary Figure 11: NusA autoinhibition can be released by λQ. (a) 1D [^{1}H,^{15}N]-HSQC and 2D [^{1}H,^{15}N]-BEST-TROSY spectra of the titration of 300 µM ^{2}H,^{15}N-NusA-SKK with NusA-AR2 (molar ratios: 1:0, black; 1:0.5, orange; 1:1, cyan; 1:2 purple; 1:5 yellow; 1:7.5 red; concentration of NusA-AR2 stock: 1.5 mM). The dashed box marks the section shown in Fig. 5a. **(b)** 1D and 2D [^{1}H,^{15}N]-HSQC spectra of the titration of 200 µM ^{15}N-NusA-AR2 with NusA-SKK (molar ratios: 1:0, black; 1:0.5, orange; 1:1, cyan; 1:2 purple; 1:5 yellow; 1:7.5 red; concentration of NusA-SKK stock: 500 µM). The dashed box corresponds to the region shown in Fig. 5b. **(c)** Determination of the affinity of the NusA-AR2:NusA-SKK interaction based on the titration of ^{15}N-NusA-AR2 with NusA-SKK. Titration curves for selected residues of ^{15}N-NusA-AR2 are shown. The individual curves representing the best fit to a two-component binding equation, yielding an average K_D value of <341 µM. **(d)** Determination of the K_D of the NusA-SKK:NusA-AR2$^{\mathrm{D443C}}$ interaction by fluorescence anisotropy measurements. 25 nM fluorescent labeled NusA-AR2$^{\mathrm{D443C}}$ was titrated with NusA-SKK. For each titration step the mean value of four individual titrations is plotted against the

NusA-SKK concentration. The standard deviation is shown as bars. The curve represents the best fit to a two-component binding equation, yielding a K_D value of 279±17 μM. **(e)** λQ removes NusA-AR2 from $^{2}H,^{15}N$-NusA-SKK. 2D [$^{1}H,^{15}N$]-BEST-TROSY spectra are shown and the dashed box indicates the section shown in Fig. 5d. Molar ratios: $^{2}H,^{15}N$-NusA-SKK:NusA-AR2:λQ =1:0, black; =1:5, orange; =1:5:1, cyan; =1:5:2, purple; =1:5:5, yellow; =1:5:10, red. Initial concentration of $^{2}H,^{15}N$-NusA-SKK: 175 μM. **(f)** λ$Q^{\Delta 36}$ detaches NusA-AR2 from $^{2}H,^{15}N$-NusA-SKK. 2D [$^{1}H,^{15}N$]-BEST-TROSY spectra are depicted, the dashed box marks the region shown in Fig. 5e. Molar ratios: $^{2}H,^{15}N$-NusA-SKK:NusA-AR2:λ$Q^{\Delta 36}$ =1:0, black; =1:5, orange; =1:5:1, cyan; =1:5:2, purple; =1:5:5, yellow; =1:5:10, red. Initial concentration of $^{2}H,^{15}N$-NusA-SKK: 175 μM.

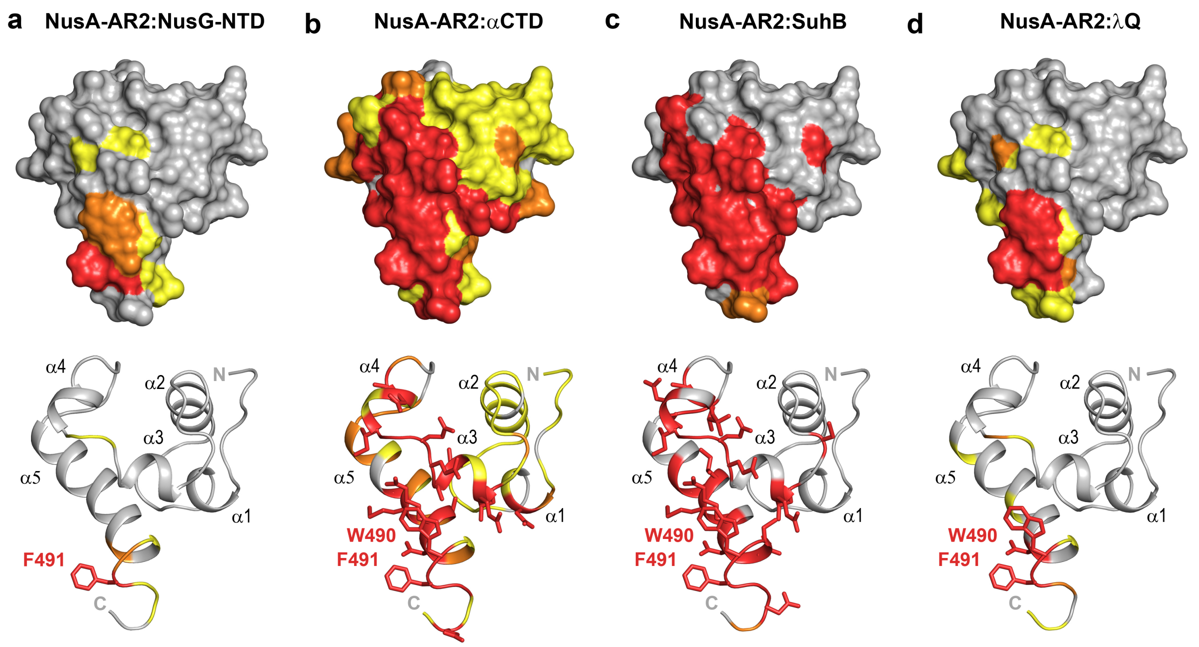

Supplementary Figure 12: NusA-AR2 interacts with various interaction partners. NusA-AR2 binding surfaces of NusG-NTD **(a)**, αCTD **(b)**, SuhB **(c)** and λQ **(d)** are shown. NusA-AR2 (gray; PDB ID: 1WCN) is in surface (top) and ribbon (bottom) representation. Residues affected by binding as determined by [^{1}H,^{15}N]-HSQC titrations are color-coded (slightly affected, yellow; moderately affected, orange; strongly affected, red). The side chains of strongly affected residues are depicted as sticks. Termini, secondary structure elements, and selected residues are labeled. Binding data for NusG-NTD, SuhB and αCTD were taken from references [1–3]. The PyMOL Molecular Graphics System (Version 1.7, Schrödinger, LLC.; https://pymol.org) was used for visualization.

Supplementary References

1. Dudenhoeffer, B. R., Schneider, H., Schweimer, K. & Knauer, S. H. SuhB is an integral part of the ribosomal antitermination complex and interacts with NusA. *Nucleic Acids Res.* **47**, 6504–6518 (2019).

2. Schweimer, K. *et al.* NusA interaction with the α subunit of E. coli RNA polymerase is via the UP element site and releases autoinhibition. *Structure* **19**, 945–954 (2011).

3. Strauß, M. *et al.* Transcription is regulated by NusA:NusG interaction. *Nucleic Acids Res.* **44**, 5971–5982 (2016).
